# Synthesis, 3D-QSAR, and Molecular Modeling Studies of Triazole Bearing Compounds as a Promising Scaffold for Cyclooxygenase-2 Inhibition

**DOI:** 10.3390/ph13110370

**Published:** 2020-11-06

**Authors:** Ranza Elrayess, Mohamed Saleh Elgawish, Marwa Elewa, Mohamed S. Nafie, Sameh S. Elhady, Asmaa S. A. Yassen

**Affiliations:** 1 Pharmaceutical Organic Chemistry Department, Faculty of Pharmacy, Suez Canal University, Ismailia 41522, Egypt; ranza.el-rayes@pharm.suez.edu.eg (R.E.); Marwa_elewa@pharm.suez.edu.eg (M.E.); 2 Medicinal Chemistry Department, Faculty of Pharmacy, Suez Canal University, Ismailia 41522, Egypt; mohamed_elgawish@pharm.suez.edu.eg; 3 Chemistry Department, Faculty of Science, Suez Canal University, Ismailia 41522, Egypt; mohamed_nafie@science.suez.edu.eg; 4 Department of Natural Products, Faculty of Pharmacy, King Abdulaziz University, Jeddah 21589, Saudi Arabia; ssahmed@kau.edu.sa

**Keywords:** triazole schiff bases scaffold, In vitro/in situ anti-inflammatory, 3D-QSAR, molecular modeling

## Abstract

Targeting of cyclooxygenase-2 (COX-2) has emerged as a powerful tool for therapeutic intervention because the overexpression of this enzyme is synonymous with inflammation, cancer, and neurodegenerative diseases. Herein, a new series of 1,2,4-triazole Schiff bases scaffold with aryl and heteroaryl systems **9a–12d** were designed, synthesized, structurally elucidated, and biologically evaluated as a potent COX-2 blocker. The rationale beyond the current study is to increase the molecule bulkiness allowing a selective binding to the unique hydrophobic pocket of COX-2. Among the triazole–thiazole hybrids, the one with the para-methoxy moiety linked to a phenyl ring **12d** showed the highest In vitro selectivity by COX-2 inhibition assay (IC_50_ of 0.04 μM) and in situ anti-inflammatory activity when evaluated using the protein denaturation assay (IC_50_ of 0.88 μM) in comparison with commercially available selective COX-2 inhibitor, Celecoxib (IC_50_ of 0.05 μM). Towards the COX-2 selectivity, ligand-based three dimensional quantitative structures activity relationship (3D-QSAR) employing atomic-based and field-based approaches were performed and resulted in the necessity of triazole and thiazole/oxazole scaffolds for COX-2 blocking. Furthermore, the molecular modeling study indicated a high selectivity and promising affinity of our prepared compounds to COX-2, especially the hydrophobic pocket and the mouth of the active site holding hydrogen-bonding, hydrophobic, and electrostatic interactions. In Silico absorption, delivery, metabolism, and excretion (ADME) predictions showed that all the pharmacokinetic and physicochemical features are within the appropriate range for human use.

## 1. Introduction

Pain is an exceedingly severe issue in 90% of illnesses, from simple back pain to pain with multiple forms of cancer. In contemporary medicine, non-steroidal anti-inflammatory drugs NSAIDs are among the most prescribed drugs. NSAIDs are very powerful to alleviate pain, fever, and inflammation and have provided relief in their use by millions of patients worldwide [1,2]. By blocking the metabolism of arachidonic acid by the cyclooxygenase enzyme (COX), these agents achieve their therapeutic effects by deteriorating the producing prostaglandin (PGs), prostacycline, and thromboxane (TXs), which play vital roles in various physiological and pathological processes [3,4]. There are three different isoforms of COX: COX-1, COX-2 and COX-3 [5]. The isoforms COX-1 and COX-2 are of primary importance, as they are involved in both physiological and pathological processes. COX-1 is expressed constitutively in a variety of cell types and is important for cytoprotective PG synthesis, pro-aggregatory thromboxaneA2 (TXA2) biosynthesis, and renal function maintenance. COX-3 is expressed only in different parts of the brain and spinal cord, and its precise roles remain unknown [5].

The induced expression of the second COX type, COX-2, is triggered by a variety of pathogenic and pro-inflammatory stimuli, such as phorbol esters, lipopolysaccharides, and cytokines. COX-2 is responsible for the biosynthesis of PGs and the development of inflammation under acute inflammatory conditions [6]. There is clear evidence stating the employment of COX-2 in various pathological conditions including inflammation, neurodegenerative diseases, and cancer. Consequently, in addition to their common use as anti-inflammatory agents, COX-2 inhibitors have recently been used for molecular and cancer imaging therapy. Therefore, the development of selective COX-2 inhibitors as anti-inflammatory and anti-cancer drugs is an important direction in pharmaceutical and academic research [7,8].

**Figure 1:**
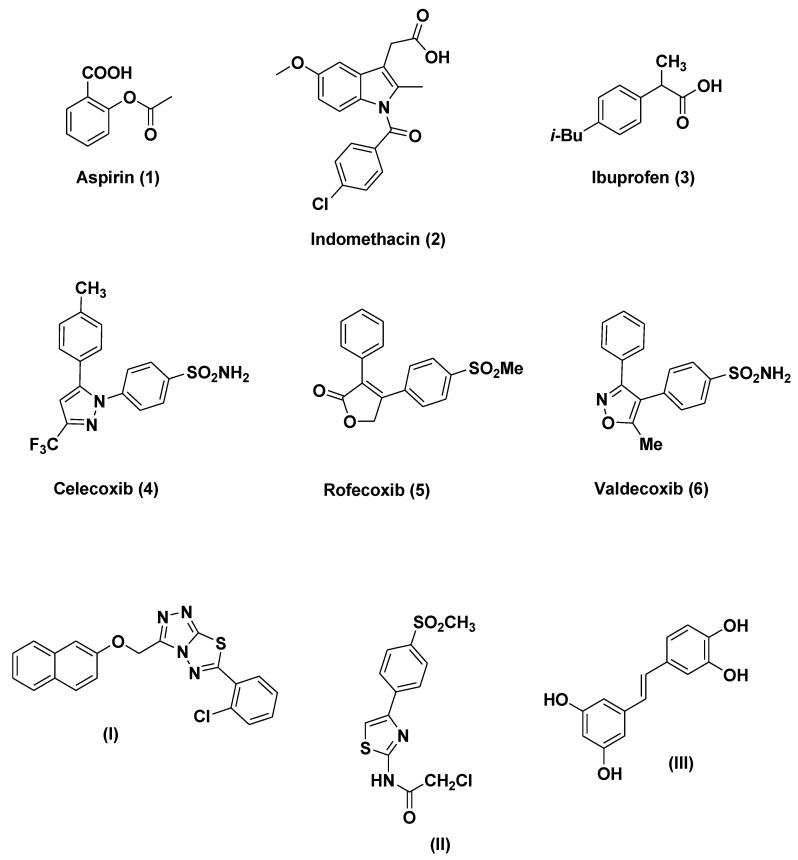
Traditional NSAIDs chemical structures; aspirin (**1**), indomethacin (**2**) and ibuprofen (**3**), selective COX-2 inhibitors; Celecoxib (**4**), rofecoxib (**5**), valdecoxib (**6**) and some reported selective Cox-2 inhibitors of diverse chemical structures (**I**–**III**).

Traditional NSAIDs such as aspirin **1**, indomethacin **2** and ibuprofen **3** (Figure 1) exert their therapeutic effect through non-selective inhibition of both COX-1 and COX-2 and in turn their use is associated with serious effects such as gastric pain, bleeding, ulcer and kidney complication [9,10]. Selective COX-2 inhibitor drugs, such as Celecoxib **4**, rofecoxib **5**, and valdecoxib **6**, have been developed in an effort to prevent these serious effects where they exhibit similar anti-inflammatory/analgesic behaviors to non-selective COX inhibitors, but with an enhanced gastric safety profile [11]. However, unfortunately, rofecoxib **5** and valdecoxib **6** have been withdrawn from the market due to their adverse changes in the biochemical COX pathway such as increased incidences of high blood pressure and myocardial infarction [12]. These side effects were attributed to the chemical structure of each drug. Therefore, the design of selective and potent anti-inflammatory drugs with enhanced safety profiles over the existing NSAIDs is still needed.

The chemical structures of reported selective COX-2 inhibitors (Figure 1) have shown a wide structural diversity and might exist in two major structural classes: tricyclics and non-tricyclics. Non-tricyclics lacked the cyclic central ring but had acyclic central 2/3 membered template systems. On the other hand, Tricyclic compounds had 1,2-diaryl substitution on a central hetero/carbocyclic ring system with a pharmacophoric group on one of the aryl rings for COX-2 selectivity [13,14,15]. Most selective COX-2 inhibitors consist of diaryl-heterocycles with a five-membered core. In drug discovery and development, heterocyclic compounds are very significant because they are involved in several biological routes. Triazole is a frequently found scaffold in a broad range of bioactive molecules including anti-microbial agents, anti-cancer, anti-viral, anti-tubercular, anti-convulsant, anti-inflammatory and analgesic, antioxidant and antidepressant properties [16]. It is recently stated that 1,2,4-triazoles and their derivatives exhibit a variety of potential therapeutic properties and given the polar structure of the triazole chain, enhances the pharmacological profile by enhancing the drug’s solubility [17]. Furthermore, the chemistry of 1, 2, 4-triazoles Schiff bases has got a great interest due to their synthetic utility and broad range biological activity including anti-microbial [18,19], anti-inflammatory [20], anti-leishmanial [21], and anti-cancer properties [22,23].

### Rational Design of Selective COX-2 Inhibitor

**Figure 2:**
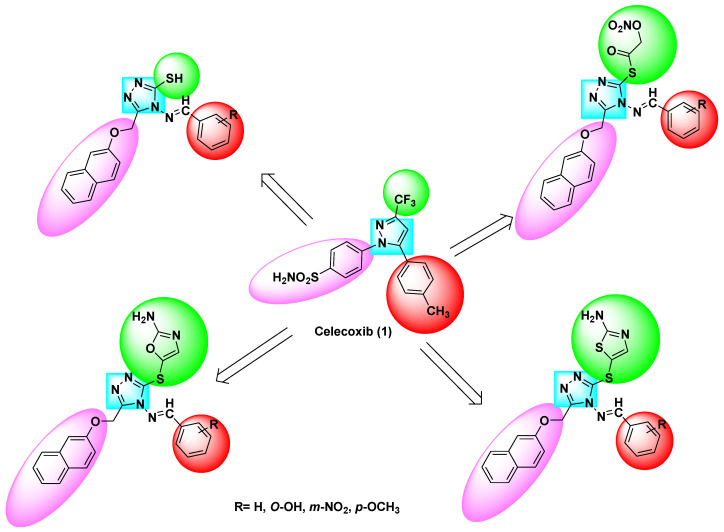
Chemical structure of the selective COX-2 inhibitor Celecoxib (1) and the designed compounds **9a–d**, **10a–d**, **11a–d**, and **12a–d**.

Developing compounds that selectively inhibit COX-2 over COX-1 is a major challenge, as both isoforms share identical positions of cell expression, amino acid composition, and molecular weight. Furthermore, the two isoforms share more than 60% of the homology and its three-dimensional structures are almost identical. The main difference though the isoleucine exchange in COX-1 for valine in COX-2 at positions 434 and 523 occurs between the COX-1 and COX-2 isozyme active sites [24]. The variation in the amino acid sequence makes the COX-2 binding site more flexible and about 25% larger by creating a separate secondary-binding pocket. Most selective COX-2 inhibitors directly bind to this secondary-binding pocket resulting in a particular COX-2 activity inhibition. Another significant area of the active COX-2 site is the hydrophobic pocket which a recent mutational research identified the use of hydrophobic pocket residues in the proper placement of fatty acid oxygenation [25,26]. Hence the highly selective and active COX-2 inhibitors should have a pharmacophore that can selectively bind into the secondary pocket exhibiting enough steric bulk to block the COX-2 hydrophobic channel. Based on the previously mentioned studies, and in continuation of our interest in the synthesis of bioactive heterocycles, herein, we describe the synthesis, in vitro evaluation as COX-1/COX-2 inhibitors, in situ anti-inflammatory activity for a new series of di/triaryl-1,2,4-triazoles Schiff bases hoping of reducing side effects with better selectivity and enhancing the anti-inflammatory activity. Moreover, molecular docking studies and 3D-QSAR of active compounds were done to get the possible binding modes of the prepared compounds into COX-2 active site and to rationalize their activity. These target compounds can be considered to be Celecoxib-like with some modifications include: (i) the replacement of the central pyrazole ring system with 1,2,4-triazole in a trial to avoid serious thromboembolic adverse effects previously reported with pyrazole derivatives [27] and to improve the drug solubility (ii) The methyl group at para position of phenyl moiety at pyrazole C3 was removed or replaced with different electronegative moieties as *m*-NO_2_, *p*-OCH_3_, *O*-OH to study the effect of various electronegative groups on COX-2 selectivity and anti-inflammatory activity. (iii) the phenyl group para-substituted with methanesulfonyl moiety (SO_2_Me) was replaced with more bulky moiety ((naphthalen-2-yloxy)methyl) which is expected to increase the interaction with the hydrophobic pocket within COX-2 active site and to improve COX-2 selectivity, as the designed compounds will be too large to fit into the smaller COX-1 active site [28] (iv) trifluoromethyl moiety (CF_3_) was replaced with different groups as SH, sulfur-linked oxazole or sulfur-linked thiazole to avoid toxicity of fluorine and to investigate various electronic effect (Figure 2). Compounds **12c** and **12d** were identified as selective and highly potent COX-2 inhibitors illustrated the feasibility of the designed rationale. Moreover, these two compounds displayed a superior in vitro anti-COX-2 and in situ anti-inflammatory activity compared to clinically administered anti-inflammatory drugs. Comprehensive computational techniques, including molecular modeling, 3D-quantitative structural activity relationship (3D-QSAR), and electronic property analysis showed that the bulkiness of the new COX-2 pharmacophore and their orientation and binding within the target protein’s binding site contribute collectively to the in situ construction of highly efficient and highly effective drug-like molecules. The description of the molecular electrostatic potentials (MESP) is a very useful tool for understanding molecular chemical reactivity and to investigate molecular electronic structure and structure–activity relationship studies. Therefore, the determination of the molecular electronic properties accountable for COX-2 inhibitors’ effective anti-inflammatory activity will shed the light on the fundamental molecular level forces responsible for the biological potency. Different electronic properties were determined, such as lowest unoccupied molecular orbital (LUMO), highest occupied molecular orbital (HOMO), and three-dimensional (3D) MESP positions. Moreover, we studied the properties of absorption, delivery, metabolism, and excretion (ADME) using physically essential descriptors and pharmaceutically specific properties to test the properties of a drug similarity to develop potent COX-2 inhibitors.

## 2. Results and Discussion

### 2.1. Chemistry

**Scheme 1:**
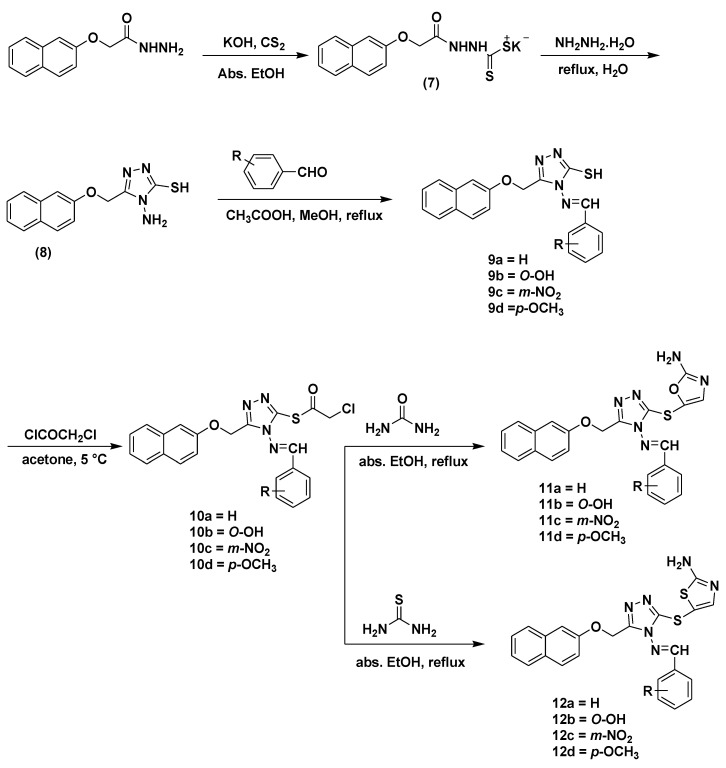
Synthesis of compounds **9a–b**, **11a–b**, **12a–b**. Abs., Absolute; EtOH, Ethanol.

A group of mercapto-1,2,4-triazole Schiff bases were synthesized using the reaction sequence illustrated in Scheme 1. The starting 2-(naphthalen-6-yloxy)acetohydrazide, was used to undergo condensation with carbon disulfide in ethanol containing potassium hydroxide to give the corresponding potassium dithiocarbamate **7**. Subsequently, the ring closure was conducted by the reaction of **7** with an excess of hydrazine hydrate to afford 4-amino-5-[(naphthalene-2yloxy)-methyl]-4*H*-1,2,4-triazole-3-thiol **8** [29]. The IR spectrum of compound **8** showed the band corresponding to SH group at range 2921–2851 cm^-1^ while ^1^H-NMR spectrum showed a characteristic single down field signal for proton of SH at range 11.08–14.22 ppm. On condensation of the mercapto-1,2,4-triazole **8** with different substituted aromatic aldehydes in methanol in the presence of glacial acetic acid furnished Schiff’s bases derivatives **9a–d** [30]. The ^1^H-NMR spectra of these compounds showed a characteristic single down field signal for proton of CH=N at range 8.30–11.64 ppm. The ^1^H-NMR spectrum of compound **9b** showed a single signal for proton of OH at 11.00 ppm while the ^1^H-NMR spectrum of compound **9d** showed a sharp upfield signal for 3 protons of OCH_3_ group at 3.81 ppm. Acylation of compounds **9a–d** was accomplished through its reaction with chloroacetyl chloride in acetone affording compounds **10a–d** [31]. Reaction of the latter compounds with urea and thiourea afforded compounds **11a–d** and compounds **12a–d** respectively [32]. The IR spectra of these compounds showed the characteristic bi forked band corresponding to NH_2_ group at range 3443–3419 cm^−1^.

### 2.2. Biological Evaluation

#### 2.2.1. In Vitro Cyclooxygenase Inhibition Assay

The in vitro COX-1/COX-2 isozyme inhibition studies measure the ability of tested compounds to inhibit bovine COX-1 and human recombinant COX-2 using an enzyme immunoassay (COX inhibitor screening assay kit, item no. 560131) according to the manufacturer’s instructions [33]. Moreover, the COX-2 selectivity indexes (S.I. values) that is defined as IC_50_ (COX-1)/IC_50_ (COX-2) were estimated and compared with that of Celecoxib as a standard drug (Table 1). The obtained data showed that the target compounds (**9a–d**, **10a–d**, **11a–d**, and **12a–d**) exhibited a wide range (moderately potent to weakly potent) of COX-1 (IC_50_ = 6.93–13.00 µM), and (moderately potent to highly potent) COX-2 (IC_50_ = 0.04–0.22 µM range, Table 1), inhibitory activities. All candidates had more potential in inhibiting COX-2 isozyme than COX-1 isozyme. The 1,2,4-triazole Schiff bases derivatives **12b–d** showed higher COX-2 potency (IC_50_ = 0.06, 0.04 and 0.04 µM respectively) and consequently higher COX-2 selectivity indexes (S.I. = 202.0, 325.0 and 311.0 respectively) than the corresponding 1,2,4-triazole Schiff bases derivatives **11a,b,d** (IC_50_ = 0.08, 0.09 and 0.06 µM respectively) and COX-2 selectivity indexes (S.I. = 154.6, 119.2 and 192.2 respectively). Within all derivatives **9a–d**, **10a–d**, **11a–d**, and **12a–d**, compound **12d** was the most potent inhibitor of COX-2 (IC_50_ = 0.04 µM) and in turn more COX-2 selective (COX-2 S.I. = 325.0) than Celecoxib (COX-2 IC_50_ = 0.05 µM, S.I. = 294.0). Furthermore, the candidate **12c** (IC_50_ = 0.0.04 µM) showed better COX-2 inhibitory activity and in turn more COX-2 selective (COX-2 S.I. = 311.8) than Celecoxib (IC_50_ = 0.05 µM).

#### 2.2.2. In Situ Anti-Inflammatory Activity

**Figure 3:**
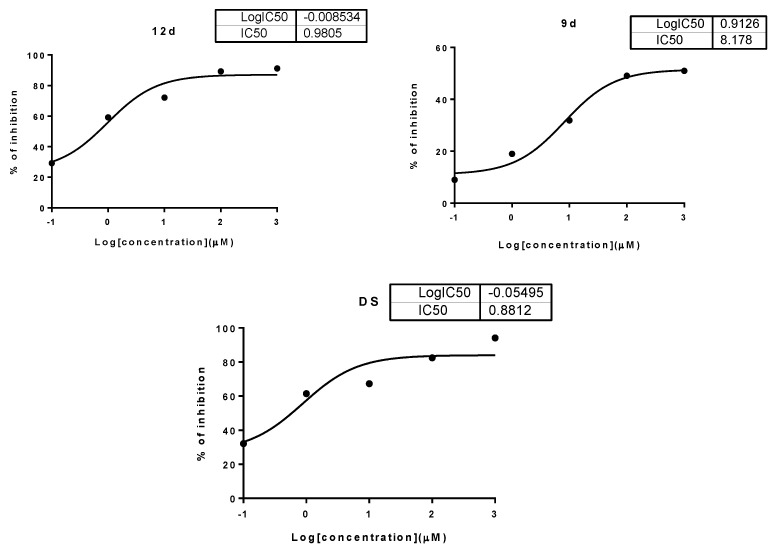
Dose-inhibition response calculation curve fit for calculation of the IC_50_ [µM] of **12d**, **9d** and Diclofenac sodium (DS) values using GraphPad prism.

Protein denaturation is used for the evaluation of anti-inflammatory activity [34]. In this methodology, generally, tertiary and secondary protein structures will typically be destroyed by the application of an external molecule or stress. The biological function of most bio-proteins is lost if it is denatured. It is well reported that a basis of inflammation is denaturation of protein. Hence, the anti-inflammatory activity was measured by the inhibition of the protein (Albumin) denaturation. As seen in Table S1 and Figure 3, compound **12d** exhibited promising anti-inflammatory activity causing 91.23% inhibition at the higher concentration 1000 µM with IC_50_ value = 0.98 µM compared to 94.15% inhibition caused by Diclofenac sodium standard control at the same concentration with IC_50_ = 0.88 µM. Meanwhile, compound **9d** exhibited week anti-inflammatory activity causing 50.99 % inhibition at the higher concentration with IC_50_ value = 8.17 µM. These results are in accordance with the results obtained from in vitro cyclooxygenase inhibition assay.

### 2.3. Computational Study

#### 2.3.1. 3D-QSAR

In this research, the model for the generation of pharmacophores was developed using Schrodinger’s pharmacophore modeling task (10.1). In the 3D-QSAR pharmacophore model, 16 1,2,4-triazole bearing compounds are considered to have promising activity against the COX-2 enzyme. A maximum of six and a minimum of five variants were selected to get the optimum feature pattern shared by the most active compounds. Phase offers a set of six pharmacophore features included: hydrogen bond acceptor (A), hydrogen bond donor (D), hydrophobic group (H), negatively charged group (N), positively charged group (P), and aromatic ring (R). Twenty pharmacophore models were produced with a specific pharmacophore combination of variants with the main feature of (AADRRR, ADRRR, AARRR). AADRRR_2 exhibited the highest alignment with the most active compounds, **12d**, between these 20 models. This variant was associated and demonstrated the best survival (4.83) site scoring (0.833), and selectivity score (2.13) and consequently it was chosen to generate the 3D-QSAR model (Table S2).

#### 2.3.2. Developing a Pharmacophore Model

**Figure 4:**
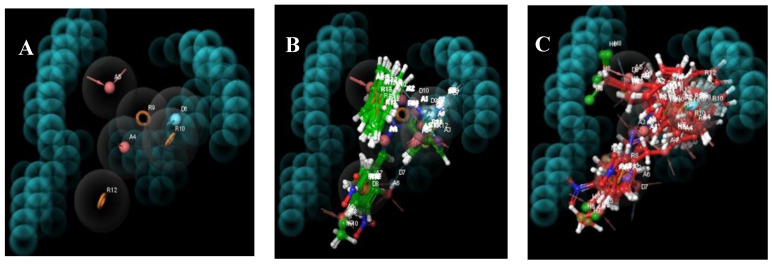
(**A**) The best generated pharmacophore model with the features considered Hydrogen bond acceptor (**A**), Hydrophobic group (H), Aromatic ring (R), (**B**) the alignment of the highly active compounds (**11a–d**, **12a–d**), and (**C**) the alignment of the less active compounds (**9a–d**, **10a–d**).

Ten pharmacophore predictive models (hypothesis) of the AADRRR type were created by aligning different conformations of the denoted training set ligands just before binding them to the developed models. At least five chemical properties were included in all the pharmacophore models produced. The model that lacks important features for ligand binding is not expected to be affordable for distinguishing between active and inactive ligands. To establish an atomic alignment of the newly synthesized compounds, the model showed the highest survival score was selected. Because of its ability to distinguish between the two active and inactive ligands in terms of atomic alignment, the AADRRR 2 model was selected. In the pharmacophore model (AADRRR_2) the particular arrangement of chemical sites or features followed by the optimum distances between them is shown in Figure 4A. The two hydrogen bonds acceptor (A) in the 12 training sets are mapped to phenyloxy oxygen and the imine moiety between the phenyl and triazole rings. The hydrogen bond donor is mainly mapped to amino group on the oxazole or thiazole moieties. On the other hand, the three aromatic ring features are mapped to naphthyl, phenyl, and triazole rings. Datasets of 12 compounds (training set) were used to produce a 3D-QSAR hypothesis based on the atom-based approach. Alignment achieved by AADRRR_2 was employed from the best model to create a 3D-QSAR. The four compounds (test set) showed the same characteristic map of the training set. The best model AADRRR_2 is clearly discriminated between the active and inactive 1,2,4-triazole bearing compounds (Figure 4B,C). The synthesized compounds lacked the amino-oxazole and amino-thiazole rings could not completely fit the model because these compounds have not the hydrogen bond donors feature and therefore these compounds (**9a–d**, **10a–d**) showed the lowest in vitro and in situ biological anti-inflammatory activity confirming the applicability of the generated pharmacophore as a promising model to discover new anti-inflammatory drug-like molecules.

#### 2.3.3. 3D-QSAR Contour Map Analysis

**Figure 5:**
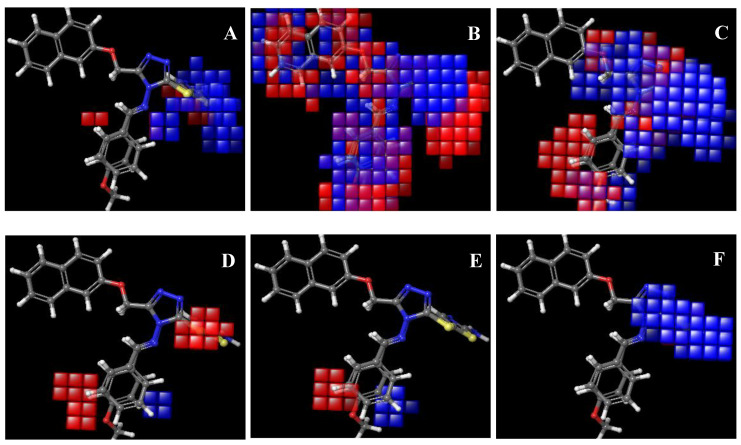
QSAR model visualized in the context of hydrogen bond donor (favorable and unfavorable) in (**A**), hydrophobic interactions in (**B**), electron-withdrawing groups in (**C**), positive ionic in (**D**), negative ionic in (**E**), and other feature in (**F**) for the highest and lowest active compounds **12d** and **9a**, respectively.

In addition to the positive and negative effects of hydrophobic/non-polar, H-bond donor, electron withdrawal, and other features mapped on the most and lowest active ligands, the created 3D-QSAR pharmacophore model is shown in Figure 5. Blue regions suggested promising features that facilitated enzyme ligand interactions, while red areas suggested unfavorable interactions are discouraging. The atomic-based QSAR contour map of H-bond donor is depicted in Figure 5A to help the comparison and visualization, the most and the least potent compounds according to in vivo activity are overlaid on the map. A big blue contour around 2-amino-thiazole moiety indicates that hydrogen bond donor is favorable for activity. This feature is restricted only for oxazole and thiazole bearing compounds (**11**, **12**). For a small red contour around thiol group in compound **9a** suggested that free thiol as H-bond donor is unfavorable and the substitution other than H is important for anti-inflammatory activity.

Moreover, the hydrophobic map of the atomic-based QSAR approach is shown in Figure 5B and to facilitate the visualization, the most and least active molecules are overlaid on the map. Blue cubes depicted the necessity of naphthyl, triazole, thiazole, and phenyl moieties for anti-COX-2 activity. However, part of the naphthyl and methoxy bridge and part of phenyl moieties are localized on the red cube meaning that there are some modifications should be done to improve the biological activity including, increasing the bridge between naphthyl and triazole core especially with hydrophilic nucleus such as oxygen and nitrogen and/or addition of certain hydrophobic group to naphthyl or phenyl to increase the hydrophobic interaction within the hydrophobic pocket of COX-2. Furthermore, the electron-withdrawing feature is concentrated around the thiazole and oxazole moieties highlighting the importance of these functional groups (Figure 5C). The amino function of oxazole and thiazole indicate that the electronegative group at this position is essential for biological activity. Additionally, there are some blue cubes were found around the ortho and meta substitution of the phenyl ring as a result of the hydroxyl and the nitro group. The compounds bearing substitution at ortho and meta positions have shown promising activity compared to unsubstituted ones. On one hand, the positive and negative ionic contour map of the highest and least active molecules are noticed mainly near the meta position of the phenyl ring revealing the importance of the nitro group for this position (Figure 5D,E). The nitro group carries both positive and negative ionic charge on nitrogen and oxygen atom, respectively. Compounds carrying a nitro group at meta position of phenyl ring tend to be more active than those without nitro group. On the other hand, the other contour map of the atomic-based QSAR approach is localized around the triazole and thiazole/oxazole moieties depicting the necessity and requirement of these moieties for potent COX-2 inhibitory activity (Figure 5F).

#### 2.3.4. Atomic-Based QSAR Validation

The generated model developed with the 3D-QSAR is valid to predict and retrieve the activity of the new molecules and the active one, respectively, if it is statistically significant. There are many rules in which the accuracy and/or usefulness of the models could be assessed. The most important statistics are the test set statistics representing by the root mean square error (RMSE), Q2, and Pearson-r, which depict how good the predictions are (Table 2). The good model should achieve an improvement in the prediction as to the number of PLS factors increases. The AADRRR_2 model was selected based on these criteria. The AADRRR_2 model was tested by predicting the activity of four synthesized compounds Table S3 and Figure S1. The stability is a marker of the model sensitivity to omission from the training set. The designed model showed promising stability which improved by increasing PLS. the ratio of the model variance to the observed activity variance could be detected by the F value. The large value of F (92.8) is a good indicator of regression statistical significance. Moreover, the data used for creating the regression model are the most suitable because of the minor value of SD of regression (0.04) and RMSE (0.24) besides the high value of R^2^ (squared correlation coefficient) (0.961). The model was also validated by the external validated correlation coefficient (Q2). The value of Q2 (0.05) is more accurate and robust than r2, since it is obtained through external validation by separation of the data into training and group test. Additionally, the value of Pearson-r (0.64) is another important parameter for depicting the correlation between the predicted and observed activity of the test set. All the data of statistical parameters are shown in Table 2. Extra validation for the produced model (AADRRR_2) was achieved by mapping two compounds with reported anti-COX-2 activity; indomethacin-dansyl conjugates [35]. The predicted values of these two compounds come in agreement with the experimental value (Table 3). The anti-COX-2 activities were predicted for these compounds and the data set was extremely similar to those experimentally measured through the created model. This fact underlines the importance of this produced model for newly synthesized compounds as a good tool for predicting anti-COX-2 activity.

#### 2.3.5. Field-Base 3D-QSAR

Field-based QSAR is a method for developing a model for the relationship between known activity values and a set of matched compounds’ 3D characteristics (like CoMFA and CoMSIA). Starting with a series of associated ligands that have known behaviors, Field-based QSAR is able to infer how the electrostatic, hydrophobic, and steric fields of the ligand contribute to biological activity or inactivity. For building and testing the model, there are two fields style could be selected: the first is a force field, which uses force-field electrostatic and steric field for the model (CoMFA) and the second field uses the five Gaussian fields for the model (CoMSIA). The green (sterically favorable) and yellow (sterically unfavorable) contours in the CoMFA steric area reflect 80% and 20% contributions at the level accordingly. Similarly, the red (electronegative charge favorable) and blue (electropositive charge favorable) contours in the CoMFA electrostatic field represent 80% and 20% level contribution, respectively.

CoMFA steric contribution contour map is shown in Figure S2A. The most and the least active compound, **12d** and **9a**, is superimposed on a map to assist the visualization. A small green contour was shown around triazole and thiazole moieties suggesting a sterically bulky group in that area preferred. The CoMFA model’s electrostatic contour map is shown in Figure S2B. The most active compound **12d** is overlaid on the map to enhance the visualization. A big red contour map at the thiazole/oxazole ring indicated that an electronegative group must present for the inhibitory activity at this position. More specifically, sulfur, oxygen, and nitrogen atoms of the heterocyclic ring are electronegative besides the amino group at position 2. Additionally, the blue contour map wrapped in the same position depicts that the electropositive group could enhance the anti-inflammatory activity. Series of compounds **11** and **12** possess the electropositive carbon of heterocyclic moieties besides the protonated amino group might be re sponsible for the high biological activity of these series compared to less active series, **9** and **10**.

In the CoMSIA hydrophobic field, the white (hydrophobic unfavorable or hydrophilic favorable) and the yellow (hydrophobic favorable) represent 10% and 90% contribution level, respectively. The CoMSIA hydrophobic contour map in the most and least active compound was described in Figure S3A, the yellow and white contour maps strike area where the hydrophobic and hydrophilic properties are favorable, respectively. The presence of a big white contour map around the ortho and meta position of the phenyl ring suggests the necessity of a hydrophilic group for biological activity. In our synthesized compounds, the substituted molecules by hydrophilic groups such as hydroxyl and nitro showed the highest activity compared to unsubstituted one. The yellow contour mainly localizes around the thiazole ring depicting that the hydrophobic moiety in this position could improve anti-inflammatory activity. Most of the highly active compounds possess thiazole and oxazole ring at this position generating a hydrophobic contour around this area. In the hydrogen bond donor fields, the purple and cyan color represent the hydrogen bond donor-favorable and unfavorable, respectively. A purple contour noticed around the thiazole/oxazole ring and near the bridge between triazole and phenyl moiety indicates that the hydrogen bond donor is favorable in these positions. In the most active compounds, the amino group is available in contrast to the lowest active compounds which lack that. Although the cyan contour seen around the methoxy bridge linked the naphthyl group to the core moiety, triazole indicates that hydrogen bond donors in this area could weaken the anti-inflammatory activity (Figure S3B).

On the other hand, in CoMSIA hydrogen bond acceptor field (Figure S3C) the red contour represents hydrogen bond acceptor favorable, while magenta represents hydrogen bond acceptor unfavorable. The red contour is seen around azo moiety which tethers the triazole core and the phenyl ring. Meanwhile, the magenta contour map is noticed near the ortho-position of the phenyl ring. In our series, we found that the presence of the hydrogen bond donor group (hydroxyl) at the ortho-position of phenyl ring gave the compound superior biological activity over the compounds lack the substitution. Hydroxyl group in ortho-position tends to be closer to red contour than magenta contour which explains the high activity of compounds **9b**, **10b**, **11b**, **12b** compared to **9a**, **10a**, **11a**, **12a**. The CoMSIA model of field-based QSAR approach can cover steric and electrostatic features (Figure S3D,E) however, the data are not shown because they are the same as CoMFA forced-field model. Extended Gaussian is another function of field-based QSAR approach which covers the same feature as Gaussian plus aromatic ring field. In the current model, the aromatic ring contour map in the presence of the most and least active compounds is shown in Figure S3E. The orange color (aromatic favorable) is seen on the thiazole/oxazole ring indicating the significance of these moieties for activity. Although the small grey contour (aromatic unfavorable) noticed in the vicinity to phenyl ring is suggesting that the presence of an aromatic ring in this position is questionable.

#### 2.3.6. Field-Base 3D-QSAR Validation

The predictive ability of the CoMFA and CoMSIA models has been calculated by the generated models of a set of 4 test compounds (Table S3). RMSE, Q2, and Pearson-r test set statistics are of importance as in atom-based QSAR because it shows how good the predictions are (Table S4). The two models had estimated the test COX-2 inhibitors correctly. The r2 values expected for the CoMFA and CoMSIA models were 0.954 and 0.958, respectively (Table S4). Moreover, the experimental and predicted pIC_50_ values for the test set compounds based on the studied CoMFA and CoMSIA models are described under 0.001 and 0.1 of the average range values, respectively (Table S3) confirming the validity of the designed model. The strong predictive power of CoMFA and CoMSIA training models for sets of various structural scaffolds indicates that these models have a large capacity for accommodation and thus broad applicability in the development of potent anti-inflammatory drug-like molecules.

### 2.4. Molecular Docking Study

**Figure 6:**
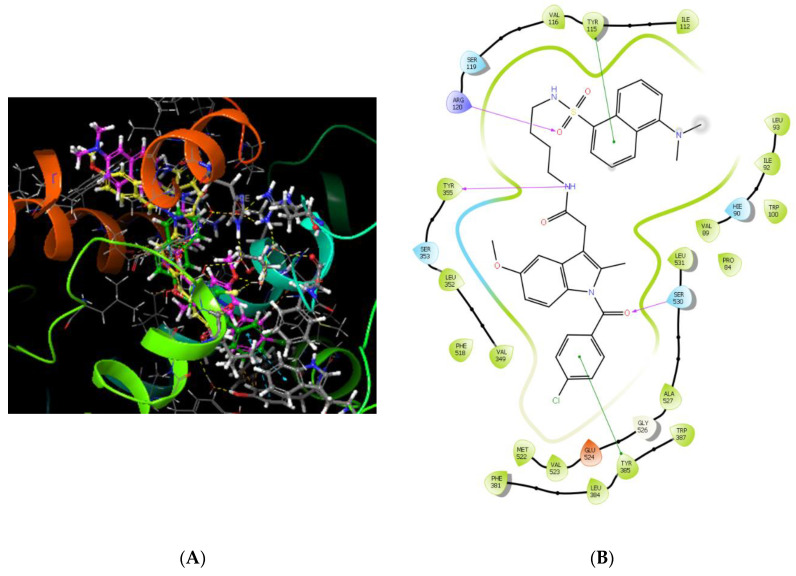

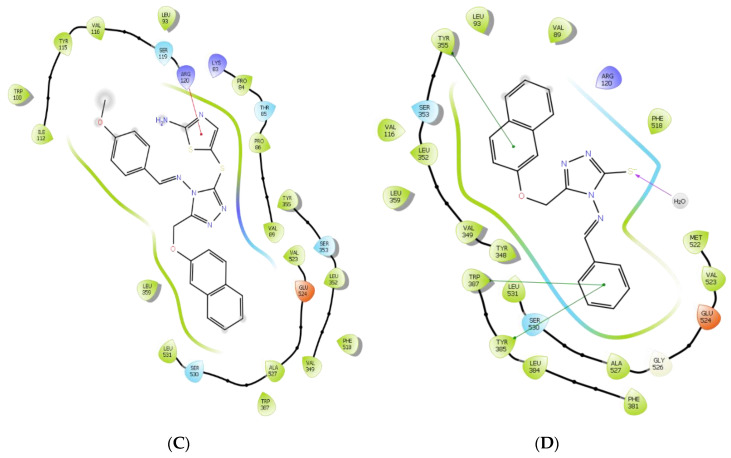
(**A**)Three-dimensional-binding modes and overlay of Dansyl-Indomethacin, compounds **9a** and **12d** in the catalytic domain of COX-2 (PDB 1M17). (**B**) 2D of dansyl-indomethacin, (**C**) 2D of compound **12d**, and (**D**) 2D of compound **9a** in COX-2 catalytic domain.

1,2,4-triazole bearing heterocyclic scaffolds were docked into the COX-2 active site to further support our experimental findings and investigate the mode of ligand-COX-2 molecular interactions. Molecular docking studies showed that hydrogen-bonding, hydrophobic and electrostatic interactions dominated the interactions of our designed compounds and COX-2 (Figure 6). It was found that thiazole and oxazole moieties deeply occupied the secondary-binding pocket region of the COX-2 active site forming hydrogen-bonding and hydrophilic/hydrophobic interactions. On the other hand, the naphthyloxy-methyl moiety was deeply inserted into the unique hydrophobic pocket of the COX-2 demonstrating hydrophobic interactions with TRP387, TYR385, PHE518, and LUE352. The binding mode of the least active compound, **9a**, is displayed in Figure 6B. The phenyl ring is deeply occupied the hydrophobic pocket surrounding by the hydrophobic residues TYR385, TRP387, LEU384, TRP348, and LUE531, forming hydrophobic interactions. The naphthyl ring is located near the opening gate of COX-2 active site forming π-π with TYR355. The 1,2,4-triazole-3-(thio/thione) moiety is located near the secondary-binding site of COX-2 forming hydrogen bond with water molecules in the same manner as indomethacin-dansyl conjugate, the ligand of the COX-2 crystal structure. The hydroxyl substitution on the ortho-position of phenyl ring potentiates drug-receptor interaction by forming extra hydrogen bond either with GLU524 or SER530, which is evident by the presence of white contour seen by the CoMSIA model. The extension of our compound by the modification at carbon three of triazole gave a new series of potent biological activity. The modification of the sulfhydryl group by chemical condensation using urea or thiourea formed oxazole and thiazole bearing amino group. This position is sterically, electrostatically, aromatically, hydrophobically, and hydrogen bond donor-favorable as evident by the studied CoMFA, and CoMSIA models. The amino groups can form a hydrogen bond with certain residues in the secondary-binding sites or near the opening gate of COX-2 including GLU524, LYS83, TYR355. Our docking study revealed the importance of hydrogen-bonding interaction between the designed drug-like molecules and the COX-2 enzyme. As evident by CoMSIA hydrogen bond donor and hydrogen bond acceptor, we could find that there are many positions that could be involved in this interaction. Hydroxyl group at the ortho-position of phenyl ring forms a hydrogen bond with GLU524 (1.59 Å), and 2-amino-thiazole/oxazole forms a hydrogen bond with TYR115 (8.7 Å) and GLU524 (1.65 Å). Similarly, N1 of triazole ring forms hydrogen bond with ARG120 (2.10 Å), and N3 of thiazole and oxazole forms hydrogen bond with TYR355 (2.54 Å). The CoMSIA hydrogen bond donor/acceptor favorable contour mainly around the thiazole/oxazole fortified the necessity of a heterocyclic system at this position. The ionic positive/negative contour of atomic-based QSAR declared the significance of nitro group at meta position of the phenyl ring, an issue confirmed by the molecular docking where nitro group forms an ionic bond with LYS83. Regarding the most potent compounds, **12d**, it was found that the naphthyl group is deeply occupied the hydrophobic pocket surrounding by TRP387, TYR385, PHE518, PHE381, and LEU352 confirming the design rationale (Figure 6C). This type of interaction gave the designed compounds superior selectivity towards COX-2 than COX-1. The molecular modeling study of the designed compounds showed that the bulkiness prevents the penetration of these compounds within the active site of COX-1; evidence confirmed by the high selectivity index in the in vitro study. The steric and hydrophobic favorable contour of the CoMFA and CoMSIA model revealed the involvement of triazole and thiazole/oxazole in these interactions. The docking study came in agreement with the QSAR pharmacophore model where the heterocyclic systems hydrophobically interact with some hydrophobic amino acid residues (TYR155, TYR355, ARG120, LYS83) near the mouth and secondary-binding site of COX-2.

A constriction created by ARG120, TYR355, and GLU524 demarcates opening into the active site. ARG120 plays an important role in stabilizing the carboxylate of classical NSAIDs however, the design compounds lack the carboxylate and thus there is no charge-charge interaction [25,26]. For instance, compound **12d** is deeply penetrated the active site leaving phenyl ring out and triazole ring near the mouth forming a hydrogen bond with TYR355, π-cation interaction with ARG120. This binding could disrupt the salt bridge between GLU524 and ARG120 leading finally to closing the mouth of the active site affecting prostaglandins biosynthesis and this is a vital issue in this study.

The best way to measure the accuracy of a docking technique is to evaluate how closely the lowest energy pose (binding conformation) predicted by the object scoring function, glide score (Gscore)/docking score in our study, resembles an experimental binding mode as calculated by the object scoring function Crystallography by X-ray. The extra-precision (XP) glide docking procedure was validated in the present study by removing the binding crystallographic indomethacin-dansyl conjugate to the COX-2 enzyme and redocking it to the binding site of the same enzyme (Figure 6D). Our research indicates excellent conformity between the location of the inhibitor from docking and the crystal structure evident by low RMSD 0.932 Å. The present study, therefore, indicates glide’s high docking reliability in the replication of the experimentally observed binding mode for COX-2 binding inhibitors. Extra-precision glide docking of compound **12d** within the active domain of COX-2 showed a reasonable docking score of −8.72 kJ/mol and a glide E-model value of −72.56 kcal mol^−1^, compared to indomethacin-dansyl conjugate that gave a docking score of −14.94 kJ/mol and a glide E-model value of −144.79 kcal mol^−1^.

### 2.5. Molecular Electrostatic Potential (MESP) and Molecular Orbital Energy Study

3D-MESP and other electronic parameters have become useful in characterizing pharmacologically active molecules. The energy calculation in Gaussian 09 applications was used for the calculation of DFT of crystallized conformation of indomethacin-dansyl conjugate and the most active compound **12d** for a well understanding of the chemical reactivity and to interpret how the polar and non-polar interaction affecting their binding to the COX-2 enzyme. The Mulliken charge distribution of the two compounds is shown in Figure S4A. The calculated atomic charge of the nitrogen atoms of triazine and oxazole/thiazole and the amino group of oxazole/thiazole is ranged from −0.534 to −0.694 suggesting their ability to involve in hydrogen bond formation. Furthermore, Mulliken atomic charges showed the negative charge (−0.187 to −0.685) of the naphthyl and phenyl ring that indicated a high electron density causing π-π interaction. The Mulliken atomic charges of the designed compound agree to that of indomethacin-dansyl conjugate which has a nitrogen atom with negative charge ranged from −0.542 to −0.674 and that of aromatic center ranged from −0.054 to −0.724.

Size and shape resemblance are apparently observed in the ESP of both compounds. The appearance of both most electropositive and most electronegative region suggest that these regions could act as electron donor or acceptor to the active site of COX-2. The most electropositive potential regions (blue color) of the most active compound are localized mainly around triazole and oxazole/thiazole moieties while the electronegative potential regions are spread over the methoxy bridge and methoxy group at the para position of the phenyl ring. The docking, CoMFA, and CoMSIA contours around these positions confirmed their involvement in the important interaction within the active site. Furthermore, the gradual depletion of red and blue colors with increasing the green-color around the aromatic center confirms the weak reactivity of these regions as evidence by the steric and hydrophobic contour of CoMFA and CoMSIA models and their hydrophobic interaction within the hydrophobic pocket of COX-2. The MESP of indomethacin-dansyl conjugate came in agreement with our most active compound (Figure S4B).

The HOMO and LUMO molecular orbital energy, associated with specific biological activities, were measured and reported for the most active compounds and indomethacin-dansyl conjugate. Mechanistically, in the ligand-protein binding, the LUMO capacity to accept electron plays a greater role than the HOMO’s electron donation. Analysis of HOMO map of compound **12d** indicates that HOMO map contour is noticed around the heterocyclic systems. The localization of HOMO molecular orbital in this position means its necessity for drug-receptor interaction and this was confirmed by the docking and pharmacophore modeling. On the other hand, the LUMO molecular orbital is localized on naphthyl moiety highlighting its rule in reactivity. When comparing the results of compound **12d** to indomethacin conjugate, it was found that there is a quite consistency between the HOMO and LUMO distribution; where the HOMO molecular orbital was noticed around the indole ring while the LUMO molecular orbital was located on dansyl moiety (Figure S5). The molecular modeling study showed the importance of these parts for ligand-enzyme interaction. Both naphthyl and dansyl moieties are deeply occupied the hydrophobic pocket of COX-2 giving these molecules superior selectivity. Furthermore, the Gaussian 09 was exploited to calculate the total energy and the energy gap between the HOMO and LUMO molecular orbital of the indomethacin -dansyl conjugate and the most active compound **12d** and found that the total energy is −2753 and −2191 Kcal mol^−1^ while the energy gaps are 0.01 and 0.05 (eV) for the indomethacin-dansyl conjugate and compound **12d**, respectively. These data confirm the stability of the two molecules and the resistance of their electron density to rearrangement under an external electrical field.

### 2.6. In Silico ADME Predictive Study

Using QikProp Schrodinger v4.3, the ADME properties were analyzed to predict the drug-like behavior of the designed compounds suggested as an active anti-inflammatory. This function provides for the evaluation of the physicochemical properties and the bioavailability assigned to the synthesized compounds based on their chemical structures. Using QikProp functionality, many pharmacokinetic parameters, such as polar surface area (PSA), QPlogS (predicted aqueous solubility), QPPCaco (predicted apparent Caco-2 cell permeability in nm s^−1^), QPPMDCK (predicted apparent MDCK cell permeability in nm s^−1^), QPlogKhsa (prediction of binding to human serum albumin), and percent human oral absorption (predicted human oral absorption on 0–100% scale) were investigated and the results depicting in Table S5. Oral activity is one of the most essential properties required in medicines. It is well known that an orally active compound should not have more than two violations of the rule of Lipinski, the thing accomplished in our established compounds confirming their drug-like properties. The oral bioavailability of the drug molecules could be regulated by rotatable (0–15) and polar bond values and PSA (7–200 Å). The 1,2,4-triazole bearing compounds revealed an excellent PSA compared to Celecoxib and diclofenac suggesting their high oral bioavailability. QPlogS and QPlogPo/w are markers of aqueous solubility and the coefficient of octanol/water partitioning; the design compounds were found to be within the permitted range. An important feature is an absorption or permeation through the intestines. Permeability of caco-2 cells (QPPCaco) is a good guide for anticipating Intestinal permeation by transportation with passive diffusion. Nearly all the design compounds have shown promising permeability regarding the commercially administered anti-inflammatory drugs. BBB permeability is a vital issue for some drugs and in some instances, such as our study, is favorable for the management of the certain neurodegenerative disorders. Most of the designed compounds showed a lower BBB permeability (QPPMDCK > 25) compared to Celecoxib and diclofenac. Another essential property of highly beneficial for the approved drugs is the long duration of the action as the dose level decreases, particularly of patients with chronic inflammation. Moreover, this feature is related to the binding to the main plasma protein, albumin. The built compounds excellently achieved this criterion compared to traditional NSAIDs. Finally, our developed compounds were seen as promising lead molecules to develop effective and selective inhibitors of COX-2 with both outstanding permeabilities of the membrane and oral bioavailability.

In tumors and inflammatory lesions, but not in normal cells, COX-2 is expressed at elevated levels. In addition, activated COX-2, including metastasis, angiogenesis, cell division, and cell death, can cause several significant steps in tumor growth. Thus, COX-2 is an ideal target for producing anti-inflammatory, anti-tumor, and anti-metastatic therapeutic agents. As both isoforms share similar positions of cell expression and amino acid composition, the development of new drugs that selectively inhibit COX-2 over COX-1 is a major challenge. The protein structures of COX-1 and COX-2 are closely conserved and have three functional domains: a growth domain analogous to an epidermal factor (N-terminal), a membrane-bound domain (MBD), and a catalytic globular domain (C-terminal). Several substrates such as NSAIDs can accommodate the active COX site, extending from the MBD to the catalytic domain interior [25]. Given many known side effects such as myocardial infarction and atherothrombotic cases, COX-inhibition-oriented medications are a billion-dollar industry that encourages scientists to look for novel COX inhibitors. A side pocket of the active COX site at the interface between the MBD and the catalytic domain, consisting of three residues (ARG120, TYR355, and GLU524) affecting the specificity of COX-1 or COX-2 substrates (Figure S6). The hydrophobic pocket is another significant area of the COX-2 active site which is lined by a group of aromatic amino acids such as tryptophan, tyrosine, and phenylalanine. Selective inhibitors might be designed to target non-conserved residues within COX-2 [24,25]. Most selective COX-2 inhibitors are diaryl-heterocycles with vicinal diaryl substituents attached to a central ring network, usually mono- or bicyclic ring. Pyrazole motif is usually preferred as a central ring because it is adapted from the first approved COX-2 inhibitor, Celecoxib, and many anti-inflammatories are bearing this ring. However, medicinal chemists are always looking for new scaffolds to investigate how the heterocyclic compounds can affect COXs inhibition [24]. In this field, the triazole scaffold was selected as the core of the proposed synthesized compounds. Triazole might improve pharmacological activity by enhancing the drug solubility an evident observed under in situ ADMT study.

Moreover, increasing the bulkiness around the triazole ring is another tactic to discover a new anti-inflammatory drug with high selectivity regarding COX-2. Incorporation of naphthyl group triggers the designed compounds to deeply occupy the unique hydrophobic pocket of COX-2 and direct the triazole ring towards the mouth of the active site and thiazole/oxazole towards the secondary-binding pocket. The disparity in the amino acid sequence makes the COX-2 substrate-binding site more flexible and about 25% larger to accommodate the designed compounds. This rationale enhances the reactivity of the proposed compounds to ARG120, TYR355, and GLU-24 forming hydrogen bond, hydrophobic, and electrostatic interaction. π-cation interaction of the triazole ring with ARG120 could disrupt the salt bridge formation between ARG120 and GLU524 leading finally to close the channel mouth. These unique interactions gave the designed compounds high selectivity and promising affinity to COX-2 over COX-1 and this is observed in the in vitro COX-2 inhibitory assay, where the 1,2,4-triazole bearing compounds showed a comparable activity and selectivity to Celecoxib. The 3D-QSAR model and the pharmacophore developing studies besides electronic and molecular electrostatic potential revealed the significance of these moieties for anti-inflammatory activity. The CoMSIA/CoMFA findings showed that a substantial inhibitory activity of a 1,2,4-triazole is attributed to the existence of hydrogen bond donor and acceptor groups besides steric and hydrophobic contour. Ligand-based drug discovery methods such as CoMFA and CoMSIA are widely used not only because they are not highly computationally intensive, but also because they can lead to the rapid generation of QSARs from which newly formed compounds can be tested for biological activity. Therefore, based on the results obtained from 3D-QSAR, various analogs of 1,2,4-triazole with certain modified feature (such as the addition of substitution at naphthyl ring, modify the methoxy and diazo methyl linker) are designed and their predictive activity (pIC_50_) was calculated using the current models. Some of the designed analogs showed enhanced pIC_50_ and high docking scores and glide energy.

## 3. Materials and Methods

### 3.1. Instrument

Melting points were measured in open capillary tubes using Stuart melting point apparatus SMP10 (UK). Infrared (IR) spectra were recorded using KBr discs on a Shimadzu Spectrophotometer (Shimadzu, Kyoto, Japan) (λmax in cm^−1^). Proton Magnetic Resonance (^1^H-NMR) and Carbon Magnetic Resonance (^13^C-NMR) were recorded using the residual solvent signal as an internal standard with a Varian AS 400 (Varian Inc., Palo Alto, CA, USA). Chemical shifts are reported in d values (parts per million, ppm) relative to tetramethylsilane (TMS) as an internal standard. Abbreviations used in NMR analysis are as follows: d = doublet, m = multiplet, q = quartet, s = singlet, t = triplet. Electron impact mass spectra (EI-MS) were recorded on DI Analysis Shimadzu QP-2010 Plus mass spectrometer. Elemental analyses were recorded on Vario EL-CHNS Elemental Analyzer (GmbH, Hanau, Germany). The results of elemental analyses (C, H, N) were found to be in good agreement (±0.45%) with the calculated values. IR, EI-MS, and elemental analyses were performed in the Microanalytical center, Cairo University, Egypt. Although ^1^H-NMR and ^13^C-NMR were performed in school of Pharmacy, Ain Shams University, Egypt. Reactions were monitored by thin layer chromatography (TLC) on Merck silica gel 60F254 and visualized with UV light. Ultrasonication was performed in ultrasound cleaner with a frequency of 50 kHz and output power of 100 W.

### 3.2. Chemicals and Reagents

Carbon disulfide, benzaldehyde, anisaldehyde, m-nitrobenzaldehyde, salicylaldehyde, chloroacetyl chloride, urea, and thiourea were purchased from Aldrich (St. Louis, MO, USA). Solvents and other reagents were of pure grade and used without further purification.

### 3.3. Experimental

#### Chemistry

**Potassium 2-(2-(naphthalen-2-yloxy)acetyl)hydrazine-1-carbodithioate (7)**

Potassium hydroxide (0.03 mol) was dissolved in absolute ethanol (50 mL). The solution was cooled in an ice bath and diphenyl acetic acid hydrazide (0.02 mol) was added to it with stirring. To this solution carbon disulfide (0.025 mol) was added in small portions with continuous stirring. The reaction mixture was continuously agitated for 13 h at RT. The precipitated potassium dithiocarbazine was filtered, washed with anhydrous ether (100 mL) and vacuum dried. The obtained potassium salt in quantitative yield was used in the next step without further purification.

**4-amino-5-((naphthalen-2-yloxy)methyl)-4*H*-1,2,4-triazole-3-thiol (8)**

A suspension of compound 7 (0.02 mol) in water (10 mL) and hydrazine hydrate (99%, 0.04 mol) was refluxed for 16 h with occasional shaking. With the evolution of hydrogen sulfide gas, the color of the reaction mixture changed to green. The progress of reaction was monitored by TLC. The reaction mixture was cooled to RT and diluted with water (20 mL). The required triazole 8 was precipitated upon acidification with acetic acid. The product was filtered, washed thoroughly with cold water, dried, and recrystallized from ethanol. (Yield 86% m.p: 177–178 °C). The ^1^H-NMR and ^13^C-NMR are as reported [15].

**General procedure for synthesis of compounds (9a–d)**

A mixture of compound 8 (0.01 mol) and appropriate aromatic aldehyde (0.01 mol) in the presence of trace amount of glacial acetic acid was refluxed for 5 h (monitored by TLC) in methanol (20 mL). The solvent was removed under reduced pressure and the product recrystallized from chloroform.

**4-(benzylideneamino)-5-((naphthalen-2-yloxy)methyl)-4*H*-1,2,4-triazole-3-thiol (9a)**

Yield (85%); m.p: 184-186 °C; Mass spectrum: m/z (%): 361 (M^+1^, 4.49%), 360 (17.89%), 340 (37.99%), 286 (100%), 271 (26.46%); IR (KBr, cm^−1^): 1219 (C-O-C), 1706 (C=N), 2921 (SH); ^1^H-NMR (DMSO, 300 MHz) δ 5.25 (s, 2H, OCH_2_), 7.25–8.40 (m, 12H, Ar-H), 11.65 (s, 1H, N=CH), 13.86 (s, 1H, SH); Anal.Calcd. for C_20_H_16_N_4_OS: C, 66.65; H, 4.47; N, 15.54; Found: C, 66.43; H, 4.63; N, 15.70; ^13^C-NMR (DMSO, 100 MHz) δ 65.27, 107.47, 119.02, 124.16, 126.87, 127.03, 127.29, 127.44 (2C), 128.02 (2C), 129.31 (2C), 129.93, 130.50, 134.47, 144.52, 156.08, 165.02, 169.44. (Figures S7 and S8)

**2-(((3-mercapto-5-((naphthalen-2-yloxy)methyl)-4*H*-1,2,4-triazol-4-yl)imino)methyl)phenol (9b)**

Yield (87 %); m.p: 208-219 °C; Mass spectrum: m/z (%): 376 (M^+^, 22.53%), 319 (29.65%), 201 (42.09%), 171 (35.63%), 81 (100%); IR (KBr, cm^−1^): 1219 (C-O-C), 1659 (C=N), 2851 (SH), 3449 (OH); ^1^H-NMR (DMSO, 300 MHz) δ 4.83 (s, 2H, OCH_2_), 6.86–7.91 (m, 11H, Ar-H), 10.00 (S, 1H, N=CH), 11.00 (s, 1H, OH) 11.88(s, 1H, SH). Anal.Calcd. for: C_20_H_16_N_4_O_2_S: C, 63.81; H, 4.28; N, 14.88; Found: C, 64.07; H, 4.39; N, 15.12. (Figure S9)

**5-((naphthalen-2-yloxy)methyl)-4-((3-nitrobenzylidene)amino)-4*H*-1,2,4-triazole-3-thiol (9c)**

Yield (81%); m.p: 200-202 °C; Mass spectrum: m/z (%): 405 (M^+^, 40.62%), 382 (100%), 362 (59.56%), 333 (55.58%), 173(91.66%); IR (KBr, cm^−1^): 1255 (C-O-C), 1294 (NO_2_), 1528 (NO_2_), 1600 (C=N), 2905 (SH); ^1^H-NMR (DMSO, 300 MHz): 5.47 (s, 2H, OCH_2_), 7.02–7.93 (m, 10H, Ar-H), 8.69 (s, IH, Ar-H (CH-NO_2_)), 10.21 (s, 1H, N=CH), 14.23 (s, 1H, SH). Anal.Calcd. for C_20_H_15_N_5_O_3_S: C, 59.25; H, 3.73; N, 17.27; Found: C, 59.44; H, 3.86; N, 17.51. (Figure S10)

**4-((4-methoxybenzylidene)amino)-5-((naphthalen-2-yloxy)methyl)-4*H*-1,2,4-triazole-3-thiol (9d)**

Yield (80%); m.p: 177–178 °C; Mass spectrum: m/z (%): 390 (M^+^, 20.07%), 326 (44.23%), 293 (17.69%), 230 (53.75%), 156 (75.72%), 105 (100%); IR (KBr, cm^−1^): 1253 (C-O-C), 1288 (OCH_3_), 1685 (C=N), 2854 (SH); ^1^H-NMR (DMSO, 300 MHz) δ 3.81(s, 3H, OCH_3_), 5.25 (s, 2H, OCH_2_), 7.00–7.99 (m, 11H, Ar-H), 8.30 (s, 1H, N=CH), 11.52 (s, 1H, SH). Anal.Calcd. for C_21_H_18_N_4_O_2_S: C, 64.60; H, 4.65; N, 14.35; Found: C, 64.83; H, 4.81; N, 14.52; ^13^C-NMR (DMSO, 100 MHz) δ 55.77, 67.42, 107.49, 114.77 (2C), 119.05, 124.10, 126.83, 127.00, 127.18, 127.94, 129.04, 129.24, 129.73, 129.89 (2C), 144.23, 148.39, 164.19, 165.63. (Figures S11 and S12)

**S-(4-(substituted benzylideneamino)-5-((naphthalen-2-yloxy)methyl)-4*H*-1,2,4-triazol-3-yl) 2-chloroethanethioate (10)**

Chloroacetyl chloride (0.05 mol) at 0–5 °C was added drop wise to a stirred solution of compounds **9a–d** (0.10 mol) in acetone (20 mL), and maintained with an ice bath for 1 h. The reaction mixture was stirred at room temperature for an additional 4 h. Then, HCl (40 mL, 10%) was added to the reaction mixture. The precipitate was formed, separated by filtration and washed with HCl (10%) and water (67%).

**General procedure for synthesis of compounds (11a–d)**

A mixture of compounds (**10a–d**) (0.02 mol) and urea (0.025 mol) in dry methanol (50 mL) was refluxed for 12 h (monitored by TLC). After the reaction completion, it was cooled and poured in to crushed ice. The solid was filtered, washed with sodium bicarbonate (2%) solution and recrystallized from ethanol.

**5-((4-(benzylideneamino)-5-((naphthalen-2-yloxy)methyl)-4*H*-1,2,4-triazol-3-yl)thio)oxazol-2-amine (11a)**

Yield (77%); m.p: 162–164 °C; Mass spectrum: m/z (%): 442 (M^+^, 20.60%), 426 (100%), 383 (73.12%), 238 (66.14%), 214 (36.65%); IR (KBr, cm^−1^): 1263 (C-O-C), 1607 (C=N), 3444-3425 (NH_2_); ^1^H-NMR (DMSO, 300 MHz) δ 5.39 (s, 2H, OCH_2_), 7.20–7.84 (m, 13H, Ar-H), 10.03 (s, 1H, N=CH); Anal.Calcd. for C_23_H_18_N_6_O_2_S: C, 62.43; H, 4.10; N, 18.99; Found: C,62.19; H,4.26; N,18.75; ^13^C-NMR (DMSO, 100 MHz) δ 65.22, 107.68, 108.48, 118.81, 118.94, 124.54, 127.04, 127.26, 128.03 (2C), 129.09 (2C), 129.38 (2C), 129.56, 129.92, 130.01, 147.50, 156.04 (2C), 162.61, 164.21, 169.29 (Figures S13 and S14).

**2-(((3-((2-aminooxazol-5-yl)thio)-5-((naphthalen-2-yloxy)methyl)-4*H*-1,2,4-triazol-4-yl)imino)methyl)phenol (11b)**

Yield (51%); m.p: 80–81 °C; Mass spectrum: m/z (%): 458 (M^+^, 29.31%), 386 (100%), 334 (47.48%), 231 (57.63%), 193 (24.88%); IR (KBr, cm^−1^): 1255 (C-O-C), 1600 (C=N), 3446-3426 (NH_2_), 3701 (OH); ^1^H-NMR (DMSO, 300 MHz) δ 4.89 (s, 2H, OCH_2_), 6.96–7.86 (m, 12H, Ar-H), 9.05 (s, 1H, N=CH); Anal.Calcd. for C_23_H_18_N_6_O_3_S: C, 60.25; H, 3.96; N, 18.33; Found:C,60.41; H,4.08; N,19.07 (Figure S15).

**5-((5-((naphthalen-2-yloxy)methyl)-4-((3-nitrobenzylidene)amino)-4*H*-1,2,4-triazol-3-yl)thio)oxazol-2-amine (11c)**

Yield (83%); m.p: 196-198 °C; Mass spectrum: m/z (%): 487 (M+, 29.17%), 479 (100%), 460 (56.31%), 399 (68.10%), 231 (67.04%); IR (KBr, cm^−1^): 1215 (C-O-C), 1351 (NO_2_), 1528 (NO_2_), 1599 (C=N), 3443-3424 (NH_2_); ^1^H-NMR (DMSO, 300 MHz) δ 5.46 (s, 2H, OCH_2_), 7.20–8.39 (m, 11H, Ar-H), 8.63 (s, IH, Ar-H(CH-NO_2_)), 10.27 (s, 1H, N=CH); Anal.Calcd. for C_23_H_17_N_8_O_4_S: C, 56.67; H, 3.52; N, 20.11; Found:C,56.89; H,3.70; N,19.89 (Figure S16).

**5-((4-((4-methoxybenzylidene)amino)-5-((naphthalen-2-yloxy)methyl)-4*H*-1,2,4-triazol-3-yl)thio)oxazol-2-amine (11d)**

Yield (39%); m.p: 70–71 °C; Mass spectrum: m/z (%): 472 (M^+^, 15.55%), 466 (30.48%), 387 (30.58%), 249 (55.46%), 57 (100%); IR (KBr, cm^−1^): 1257 (C-O-C), 1384 (OCH_3_), 1658 (C=N), 3452–3419 (NH_2_); ^1^H-NMR (DMSO, 300 MHz) δ 3.82(s, 3H, OCH_3_), 5.36 (s, 2H, OCH_2_), 6.97–7.84 (m, 12H, Ar-H), 9.50 (s, 1H, N=CH); Anal.Calcd. for C_24_H_20_N_6_O_3_S: C, 61.01; H, 4.27; N, 17.79; Found: C,60.95; H,4.40; N,17.86; ^13^C-NMR (DMSO, 100 MHz) δ 55.59, 66.59, 107.82, 114.81 (2C), 118.02, 119.11, 124.40, 126.97, 127.26, 128.00, 129.40, 129.52, 129.60, 129.81, 130.02 (2C), 134.48, 135.00, 150.03, 156.01, 157.00, 162.09, 167.29 (Figures S17 and S18).

**General procedure for synthesis of compounds (12a–d)**

A mixture of compounds (**10a–d**) (0.02 mol) and thiourea (0.025 mol) in absolute ethanol (50 mL) was refluxed for 12 h (monitored by TLC). After the reaction completion, it was cooled and poured onto crushed ice. The solid was filtered, washed with sodium bicarbonate (2%) solution and recrystallized from ethanol.

**5-((4-(benzylideneamino)-5-((naphthalen-2-yloxy)methyl)-4*H*-1,2,4-triazol-3-yl)thio)thiazol-2-amine (12a)**

Yield (75%); m.p: 145-146 °C; Mass spectrum: m/z (%): 460 (M^+2^, 11.38%), 458 (37.02%), 424 (18.82%), 396 (29.39%), 370 (97.25%), 77 (100%); IR (KBr, cm^−1^): 1256 (C-O-C), 1603 (C=N), 3443-3423 (NH_2_); ^1^H-NMR (DMSO, 300 MHz) δ 4.90 (s, 2H, OCH_2_), 7.23–7.86 (m, 13H, Ar-H), 10.03 (s, 1H, N=CH); Anal.Calcd. for C_23_H_18_N_6_OS_2_: C, 60.24; H, 3.96; N, 18.33; Found:C,60.13; H,4.18; N,18.50 (Figure S19).

**2-(((3-((2-aminothiazol-5-yl)thio)-5-((naphthalen-2-yloxy)methyl)-4*H*-1,2,4-triazol-4-yl)imino)methyl)phenol (12b)**

Yield (47%); m.p: 77-78 °C; Mass spectrum: m/z (%): 474 (M^+^, 12.34%), 358 (90.24%), 339 (79.05%), 270 (100%), 234 (54.89%); ^1^H-NMR (DMSO, 300 MHz) δ 4.90 (s, 2H, OCH_2_), 6.96–7.85 (m, 12H, Ar-H), 9.0 1(s, 1H, N=CH); Anal.Calcd. for C_23_H_18_N_6_O_2_S_2_: C, 58.21; H, 3.82; N, 17.71; Found: C,58.47; H,4.05; N,17.89 (Figure S20).

**5-((5-((naphthalen-2-yloxy)methyl)-4-((3-nitrobenzylidene)amino)-4*H*-1,2,4-triazol-3-yl)thio)thiazol-2-amine (12c)**

Yield (79%); m.p: 182-184 °C; Mass spectrum: m/z (%): 503 (M+, 20.74%), 392 (24.17%), 461 (41.43%), 299 (43.93%), 44 (100%); IR (KBr, cm^−1^): 1257 (C-O-C), 1319 (NO_2_), 1599 (NO_2_), 1685 (C=N), 3444–3422 (NH_2_); ^1^H-NMR (DMSO, 300 MHz) δ 5.46 (s, 2H, OCH_2_), 7.20–8.88 (m, 11H, Ar-H), 8.63 (s, IH, Ar-H(CH-NO_2_)), 10.30 (s, 1H, N=CH); Anal.Calcd. for C_23_H_17_N_7_O_3_S_2_: C, 54.86; H, 3.40; N, 19.47; Found: C,55.12; H,3.67; N,19.51 (Figure S21).

**5-((4-((4-methoxybenzylidene)amino)-5-((naphthalen-2-yloxy)methyl)-4*H*-1,2,4-triazol-3-yl)thio)thiazol-2-amine (12d)**

Yield (41%); m.p: 69-70 °C; Mass spectrum: m/z (%): 488 (M^+^, 13.31%), 463 (23.21%), 436 (41.80%), 357 (100%), 326 (68.18%); IR (KBr, cm^−1^): 1269 (C-O-C), 1384 (OCH_3_), 1630 (C=N), 3424-3419 (NH_2_); ^1^H-NMR (DMSO, 300 MHz) δ 3.81 (s, 3H, OCH_3_), 5.36 (s, 2H, OCH_2_), 7.19–7.86 (m, 12H, Ar-H) 9.85 (s, 1H, CH); Anal.Calcd. for C_24_H_20_N_6_O_2_S_2_: C, 59.00; H, 4.13; N, 17.20; Found: C,59.23; H,4.28; N,17.03 (Figure S22).

### 3.4. Biological Assays

#### 3.4.1. Cyclooxygenase Inhibition Assays

The ability of the test compounds **9a–d**, **11a–d** and **12a–d** listed in Table 1 to inhibit bovine COX-1 and human recombinant COX-2 (IC_50_ value, µM) was assigned using an enzyme immune assay (EIA) kit (item no. 560131, Cayman Chemical, Ann Arbor, MI, USA) according to the previously reported method [33].

#### 3.4.2. In Situ Anti-Inflammatory Assay

Protein denaturation method was proposed by (Mizushima and Kobayashi, 1968) [34] with certain modifications has been used to evaluate the anti-inflammatory (in vitro) activity [34]. The reaction mixture is molecules with serial dilutions (1000, 100, 10, 1, 0.1 µM), phosphate buffered saline (PBS; pH 6.4), and egg albumin. The control used was double distilled water (equal volume). These samples were incubated at 37 °C for 15 min and then heated at 70 °C for 5 min. in a water bath. After attaining ambient temperature, the absorbance of samples was measured spectrophotometrically at 660 nm using vehicle as blank. Diclofenac sodium was taken as the standard drug. The experiment was performed in triplicate. Percent inhibition of protein denaturation was calculated using the Equation Inhibition (%) of protein denaturation inhibition (%) = (A_C_ − A_S_)/A_C_ × 100 where A_C_: absorbance of control, A_S_: absorbance of sample.

### 3.5. Molecular Modeling Study

#### 3.5.1. QSAR

A set of 20 compounds containing 1,2,4-triazole scaffolds was synthesized and selected to form the QSAR model. The inhibition potentialities of the target compounds in data set were identified as IC_50_ values ranged from 25 to 58 nM. The IC_50_ values were converted to molar values, which were then converted to pIC_50_ values using the formula below:

pIC_50_ = −Log (IC_50_).

The builder’s panel in Maestro used to construct the 3D structures of 1,2,4-triazole derivatives. Such structures have also been optimized using the LigPrep module (v2.1; Schrodinger 2015-1, New York, NY, USA) as mentioned below [36].

#### 3.5.2. Pharmacophore 3D-QSAR Modeling

Phase (v4.1; Schrodinger 2015-1) was used for the generation of COX-2 receptor pharmacophores and 3D-QSAR models. For the creation of the pharmacophore phase model the prepared ligands with their respective biological activity values, pIC_50_, were imported. The ligands were allocated to be active with a pIC_50_ > 1.0 threshold, and inactive with a pIC_50_ < 1.0 threshold. The residual compounds were considered to be moderately active. Phase (v4.0) type screening was used in the present study for versatile alignment of the identified COX-2 inhibitors using the highest active **12d** compound as a template. Using the established default, one hundred conformers were produced with no more than 10 conformations per rotatable bond. The most critical and difficult step is to pick the training and test sets. A commonly used methodology and a random division were applied to determine robustness of the QSAR model. There were three trials underway in this process. Pharmacophore sites for 1,2,4-triazole training and testing included the usual range of phase chemical characteristics: one hydrogen bond acceptor (A), one hydrogen donor (D), hydrophobic interaction (H), and two aromatic rings (R) [36].

#### 3.5.3. Model Validation

The QSAR model AADRRR_2 was described as the best model, with six components PLS variables. The predicted COX-2 inhibition of training set ligands behavior provoked a 0.85 correlation (R) with the observed inhibition. Template AADRRR_2’s efficacy was also tested on external validation. It also plotted a graph of the real value versus the expected value [36].

#### 3.5.4. Molecular Modeling Study

In the current work, a study of molecular modeling was carried out using the glide docking method implemented in the molecular modeling software (Schrodinger-10.1). The X-ray crystal structure of COX-2 enzyme in complex with dansyl-indomethacin conjugate (PDB ID:6BL3 resolution 2.22 Å) was obtained from (PDB; http://www.rcsb.org/pdb). Full procedures regarding protein preparation, ligands optimization, software validation are supported at the supplementary part as previously published [37,38].

### 3.6. Computational Study

All DFT computations were carried out using Gaussian 09 software (Gauss View 5.0, Gaussian, Inc., Wallingford, CT, USA). Hybrid density functional theory with Becke’s three-parameter exchange potential and the Lee–Yang–Parr correlation functional (B3LYP), using the basis set 3–21 G* level was carried out for complete geometry optimization [39]. The quantum chemical descriptors, including Mulliken charge distribution, MESP, HOMO, and LUMO were computed using Jaguar. 3D isosurfaces of the MESPs at the van der Waals contact surface represent electrostatic potentials superimposed onto a surface of constant electron density.

### 3.7. Lipinski’s Rule for Drug Likeliness and In Silico ADME Prediction

In accordance with Lipinski’s rule of five ADME, drug-like properties of newly synthesized compounds with anticipated biological and/or pharmacological activity were evaluated, which was used to evaluate if these compounds have the properties that would allow them to be a possibly orally active drug for humans. The drug-like action of our compounds has been predicted using module QikProp (v4.2; Schrodinger 2015-1). For the measurement of pharmacokinetic parameters by QikProp v4.2, the 1,2,4-triazole compounds prepared by the LigPrep module v3.1; Schrodinger 2015-1 and were used as mentioned [40].

## 4. Conclusions

In this study, Schiff-based triazole bearing heterocyclic systems were designed, synthesized, structurally elucidated, and biologically evaluated as a potent and selective COX-2 inhibitor. The ligand-based 3D-QSAR approach using CoMFA and CoMSIA tool revealed the importance hydrogen bond donor/acceptor beside steric and hydrophobic field to molecule-COX-2 interaction. Docking study as well as computational study including molecular orbital HOMO/LUMO, molecules, total energy, and electrostatic surface potential also exploited to identify the key feature crucial for binding mechanism. The comprehensive QSAR and computational studies predicted that the bulkiness of the designed molecules, aryl and heteroaryl pharmacophores, and the proper orientation of these moieties inside the hydrophobic pocket and near the channel mouth collectively contribute to In vitro and in situ anti-inflammatory activity. Furthermore, the findings of 3D-QSAR model were found to be reliable to predict COX-2 blocking activity of diverse structural molecules and to improve the optimization of new triazole derivatives. Briefly, the findings of this study provide a cost-effective and quick screening tools for the drug-like candidates’ discovery. Currently, we extend working on the designed scaffold to expand its reactivity towards lipoxygenase.

## Figures and Tables

**Table 1:** In vitro COX-1 and COX-2 inhibition of mercapto-1,2,4-triazole Schiff bases derivatives (**9a–d**, **11a–d**, **12a–d**) and Celecoxib.

Compound No.	IC_50_ (µM) ^a^		
	COX-1	COX-2	COX-2 S.I. ^b^
**9a**	6.93	0.22	31.5
**9b**	7.67	0.19	40.4
**9c**	9.43	0.13	72.5
**9d**	7.97	0.15	53.1
**10a**	9.72	0.13	74.8
**10b**	9.89	0.14	70.6
**10c**	10.12	0.12	84.3
**10d**	10.35	0.11	94.1
**11a**	12.37	0.08	154.6
**11b**	10.73	0.09	119.2
**11c**	12.63	0.05	252.6
**11d**	11.53	0.06	192.2
**12a**	9.83	0.1	98.3
**12b**	12.13	0.06	202.2
**12c**	12.47	0.04	311.8
**12d**	13	0.04	325.0
Celecoxib	14.7	0.05	294.0

^a^ IC_50_ value represents the compound concentration that is required to produce 50% inhibition of COX-1 or COX-2 which is the mean value of two determinations where the deviation from the mean is <10% of the mean value. ^b^ Selectivity index (COX-1 IC_50_/COX-2 IC_50_).

**Table 2:** PLS statistical parameters of the model AADRRR_2.

PLS	SD	R2	R2 CV	Stability	F	P	RMSE	Q2	Pearson-r
**1**	0.1018	0.7786	0.2547	0.714	45.7	1.34 × 10^−5^	0.27	−0.19	0.5598
**2**	0.0555	0.9393	0.1111	0.202	92.8	5.01 × 10^−8^	0.24	0.0566	0.639
**3**	0.0464	0.9611	0.1983	0.242	90.6	4.86 × 10^−8^	0.25	−0.0618	0.6058

**Table 3:** Calculated pIC_50_ for the designed and the reported compounds.

In	Ligand Name	QSAR Set	Activity	Predicted Activity	Activity	Prediction Error
**1**	9a	training	0.657	0.753	Inactive	0.096
**2**	9b	training	0.721	0.741	Inactive	0.02
**3**	9c	training	0.886	0.809	Inactive	−0.076
**4**	9d	training	0.824	0.787	Inactive	−0.036
**5**	10a	test	0.886	1.011	Inactive	0.125
**6**	10b	training	0.853	0.884	Inactive	0.031
**7**	10c	training	0.92	0.92	Inactive	0.002
**8**	10d	training	0.958	0.97	Inactive	0.012
**9**	11a	training	1.097	1.097	Active	0.001
**10**	11b	training	1.046	1.046	Active	0.001
**11**	11c	test	1.31	1.101	Active	−0.208
**12**	11d	training	1.221	1.216	Active	−0.004
**13**	12a	training	1	0.989	Active	−0.01
**14**	12b	test	1.221	1.019	Active	−0.201
**15**	12c	training	1.397	1.396	Active	0
**16**	12d	test	1.397	0.973	Active	−0.423
**17**	Dansyl-Indomethacin (1)	test	0.77	0.700		−0.069
**18**	Dansyl-Indomethacin (2)	test	0.12	0.127		0.007

## Supplementary Materials

The following are available online at https://www.mdpi.com/1424-8247/13/11/370/s1. Table S1: Anti-inflammatory activity of compounds **12d**, **9d** compared to Diclofenac sodium (DS) using protein denaturation method. Table S2. Score of different parameters of the obtained hypotheses. Table S3. Calculated pIC50 for designed compounds. Table S4. PLS statistical parameters of the designed Field-base model. Table S5. *In silico* ADME prediction parameters of designed and reference molecules. Figure S1. Scatter plot of the observed versus phase-predicted activity for (a) training set and (b) test set compounds with best fit line. Figure S2. Field-based CoMFA model visualized in the context of favorable and unfavorable Steric (A) and Electrostatic (B) for the highest and lowest active compounds **12d** and **9a**, respectively. Figure S3. Field-based CoMSIA model visualized in the context of favorable and unfavorable Hydrophobic (A), Hydrogen bond donor (B), Hydrogen bond acceptor (C), Steric (D), Electrostatic (E), Aromatic (F) for the highest and lowest active compounds **12d** and **9a**, respectively. Figure S4. (a) Mulliken charges, (b) electrostatic surface potential calculated using 3-21G* (d,p) basic set methodology (color-coded from red to blue) and density functional theory method with B3LYP functional. Figure S5. Plots of HOMO and LUMO of compound **12d** on left side and dansyl-indomethacin on right side. Figure S6. The binding site of COX-2 enzyme shows the secondary-binding pocket in blue and the unique hydrophobic pocket in yellow. Figure S7. 1H-NMR spectrum of compound **9a**. Figure S8. 13C-NMR spectrum of compound **9a**. Figure S9. 1H-NMR spectrum of compound **9b**. Figure S10. 1H-NMR spectrum of compound **9c**. Figure S11. 1H-NMR spectrum of compound **9d**. Figure S12. 13C-NMR spectrum of compound **9d**. Figure S13. 1H-NMR spectrum of compound **11a**. Figure S14. 13C-NMR spectrum of compound **11a**. Figure S15. 1H-NMR spectrum of compound **11b**. Figure S16. 1H-NMR spectrum of compound **11c**. Figure S17. 1H-NMR spectrum of compound **11d**. Figure S18. 13C-NMR spectrum of compound **11d**. Figure S19. 1H-NMR spectrum of compound **12a**. Figure S20. 1H-NMR spectrum of compound **12b**. Figure S21. 1H-NMR spectrum of compound **12c**. Figure S22. 1H-NMR spectrum of compound **12d**.
