# Supplementary material for: Synthesis, 3D-QSAR, and Molecular Modeling Studies of Triazole Bearing Compounds as a Promising Scaffold for Cyclooxygenase-2 Inhibition
Source: Pharmaceuticals (Basel). 2020 Nov 6;13(11):370. doi: 10.3390/ph13110370 (PMC7694773; doi:10.3390/ph13110370)
Supplement: Supplementary file 1 [file pharmaceuticals-13-00370-s001.pdf]

Synthesis, 3D-QSAR and Molecular Modeling Studies of Triazole Bearing Compounds as a Promising Scaffold for Cyclooxygenase-2 Inhibition

Ranza Elrayess[1], Mohamed Saleh Elgawish[2], Marwa Elewa1, Mohamed S. Nafie[3], Sameh S. Elhady[4], Asmaa S. A. Yassen[1,*]

1 Pharmaceutical Organic Chemistry Department, Faculty of Pharmacy, Suez Canal University, Ismailia (41522), Egypt; ranza_elrayess@yahoo.com (R.E); Marwa_elewa@pharm.suez.edu.eg (M.E) ; asmaa_yaseeen@pharm.suez.edu.eg (A.S.A.Y)

2 Medicinal Chemistry Department, Faculty of Pharmacy, Suez Canal University, Ismailia (41522), Egypt, mohamed_elgawish@pharm.suez.edu.eg

3 Chemistry Department, Faculty of Science, Suez Canal University, Ismailia (41522), Egypt, mohamed_nafie@science.suez.edu.eg

4 Department of Natural Products, Faculty of Pharmacy, King Abdulaziz University, Jeddah (21589), Saudi Arabia, ssahmed@kau.edu.sa

*Correspondence: asmaa_yaseeen@pharm.suez.edu.eg; Tel.: +20-1096206738; Fax: +20-064-3230741

Additional Experimental Details

1) Table S1: Anti-inflammatory activity of compounds 12d, 9d compared to Diclofenac sodium (DS) using protein denaturation method
2) Table S2. Score of different parameters of the obtained hypotheses
3) Table S3. Calculated pIC_{50} for designed compounds
4) Table S4. PLS statistical parameters of the designed Field-base model
5) Table S5. In *silico* ADME prediction parameters of designed and reference molecules.
6) Figure S1. Scatter plot of the observed versus phase-predicted activity for (a) training set and (b) test set compounds with best fit line.
7) Figure S2. Field-based CoMFA model visualized in the context of favorable and unfavorable Steric (A) and Electrostatic (B) for the highest and lowest active compounds 12d and 9a, respectively.
8) Figure S3. Field-based CoMSIA model visualized in the context of favorable and unfavorable Hydrophobic (A), Hydrogen bond donor (B), Hydrogen bond acceptor (C), Steric (D), Electrostatic (E), Aromatic (F) for the highest and lowest active compounds 12d and 9a, respectively.
9) Figure S4 (a) Mulliken charges, (b) electrostatic surface potential calculated using 3-21G* (d,p) basic set methodology (color-coded from red to blue) and density functional theory method with B3LYP functional
10) Figure S5. Plots of HOMO and LUMO of compound 12d on left side and dansyl-indomethacin on right side.
11) Figure S6. The binding site of COX-2 enzyme shows the secondary binding pocket in blue and the unique hydrophobic pocket in yellow.
12) Figure S7. 1H-NMR spectrum of compound 9a
13) Figure S8. ^{13}C-NMR spectrum of compound 9a
14) Figure S9. ^{1}H-NMR spectrum of compound 9b
15) Figure S10. ^{1}H-NMR spectrum of compound 9c

16) Figure S11. ^{1}H-NMR spectrum of compound 9d
17) Figure S12. ^{13}C-NMR spectrum of compound 9d
18) Figure S13. ^{1}H-NMR spectrum of compound 11a
19) Figure S14. ^{13}C-NMR spectrum of compound 11a
20) Figure S15. ^{1}H-NMR spectrum of compound 11b
21) Figure S16. ^{1}H-NMR spectrum of compound 11c
22) Figure S17. ^{1}H-NMR spectrum of compound 11d
23) Figure S18. ^{13}C-NMR spectrum of compound 11d
24) Figure S19. ^{1}H-NMR spectrum of compound 12a
25) Figure S20. ^{1}H-NMR spectrum of compound 12b
26) Figure S21. ^{1}H-NMR spectrum of compound 12c
27) Figure S22. ^{1}H-NMR spectrum of compound 12d

Table S1. Anti-inflammatory activity of compounds 12d, 9d compared to Diclofenac sodium (DS) using protein denaturation method.

Comp. No.	% of inhibition± SEM* at conc.					IC_{50}[#] (μM)
	0.1	1	10	100	1000	
12d	29.21±1.12	59.20±1.34	72.12±1.27	89.31±1.26	91.23±2.10	0.98
9d	8.97±0.97	18.95±1.04	31.86±0.96	49.08±1.05	50.99±1.08	8.178
DS	32.14±1.01	61.45±1.21	67.32±0.95	82.43±1.87	94.15±2.01	0.88

*Values are expressed as Mean±SEM of three replicates. [#]IC_{50} values are calculated using non-linear regression curve fit of sigmoidal dose-inhibition response using GraphPad prism7 software.

Table S2. Score of different parameters of the obtained hypotheses.

Site Types	Survival Score	Site Score	Volume Score	Selectivity Score	Num Matched	Inactive Score	Adjusted Score
AADRRR_2	4.835	0.833	0.873	2.129	10	0.654	4.181
ADRRR_7	4.42	0.835	0.862	1.723	10	0.968	3.452
AARRR_1	4.429	0.88	0.867	1.682	10	1.305	3.124
ADRRR_2	4.459	0.824	0.873	1.762	10	0.621	3.837
AADRRR_1	4.459	0.824	0.873	1.762	10	0.621	3.837
AADRRR_3	4.832	0.822	0.866	2.144	10	0.71	4.122
ADRRR_4	4.439	0.847	0.855	1.737	10	0.988	3.451
AADRRR_6	4.821	0.819	0.86	2.143	10	1.029	3.792
AADRRR_8	4.811	0.823	0.864	2.124	10	0.556	4.256
ADRRR_1	4.465	0.852	0.855	1.758	10	1.161	3.303
AADRRR_5	4.829	0.791	0.877	2.161	10	0.514	4.314
ADRRR_9	4.415	0.775	0.875	1.765	10	0.687	3.728
ADRRR_8	4.416	0.805	0.868	1.742	10	0.781	3.635
ADRRR_6	4.425	0.803	0.869	1.753	10	0.709	3.716
ADRRR_3	4.44	0.815	0.852	1.773	10	1.041	3.399
AADRRR_4	4.83	0.822	0.866	2.142	10	0.511	4.319
AADRRR_9	4.81	0.767	0.876	2.167	10	0.529	4.281
AADRRR_7	4.813	0.801	0.86	2.152	10	0.532	4.281
ADRRR_5	4.426	0.812	0.862	1.752	10	0.616	3.81
AADRRR_10	4.809	0.793	0.869	2.147	10	0.5	4.309

Table S3. Calculated pIC_{50} for designed compounds.

In	Ligand Name	QSAR Set	Activity	Predicted Activity	Activity	Prediction Error
1	9a	training	0.658	0.762	Inactive	0.105
2	9b	training	0.721	0.735	Inactive	0.014
3	9c	training	0.886	0.809	Inactive	-0.077
4	9d	training	0.824	0.798	Inactive	-0.026
5	10a	test	0.886	1.047	Inactive	0.138
6	10b	training	0.854	0.892	Inactive	0.038
7	10c	training	0.921	0.922	Inactive	0.001
8	10d	training	0.959	0.946	Inactive	-0.013
9	11a	training	1.097	1.086	Active	-0.011
10	11b	training	1.046	1.043	Active	-0.002
11	11c	test	1.301	1.074	Active	-0.227
12	11d	training	1.221	1.231	Active	0.010
13	12a	training	1	1.006	Active	0.006
14	12b	test	1.221	1.050	Active	-0.171
15	12c	training	1.397	1.391	Active	-0.006
16	12d	test	1.379	0.979	Active	-0.400

Table S4. PLS statistical parameters of the designed Field-base model.

PLS	SD	R^2	R^2 CV	Stability	F	P	RMSE	Q2	Pearson-r
1	0.1016	0.7788	0.4041	0.828	45.8	1.33E-05	0.29	-0.4192	0.3405
2	0.0587	0.9317	0.2939	0.436	81.9	1.01E-07	0.23	0.0436	0.6037
3	0.0503	0.9542	0.3748	0.457	76.4	1.19E-07	0.24	-0.0277	0.5871

Table S5. *In Silico* ADME prediction parameters of designed and reference molecules.

ADME Prediction Parameters	**Compound 12d**	**Reference NSAIDs**	
		**Diclofenac**	**Celecoxib**
mol MW[a]	493	296.152	381.372
Donor-HB[b]	**2**	**2**	**2**
Accept-HB[c]	**6.25**	**2.5**	**5.5**
QPlogPo/w[d]	**5.8**	4.502	3.337
PSA[e]	87.837	81.267	57.807
QPlogS[f]	-7.962	-5.331	-5.825
QPPCaco[g]	446.611	358.155	355.215
QPlogBB[h]	-1.258	-0.2	-0.776
QPPMDCK[i]	1410.623	781.46	787.429
QPlogKhsa[j]	0.883	0.041	0.366
% Human Oral Absorption[k]	95.4	100	92.1

Note: Acceptable ranges: a<500 amu; b<5; c<10; d<5; e7–200; f<0.5; g≤5; h<25 poor, >500 great; i<25 poor, >500 great; j−1.5 to 1.5; k>80% is high, <25% is poor.

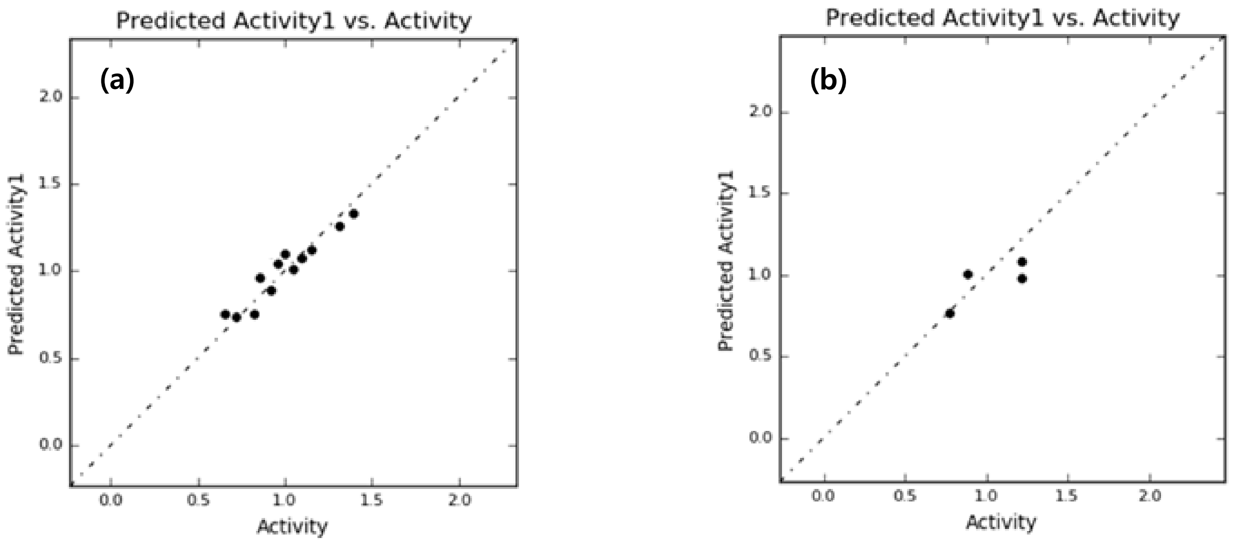

Figure S1. Scatter plot of the observed versus phase-predicted activity for (a) training set and (b) test set compounds with best fit line.

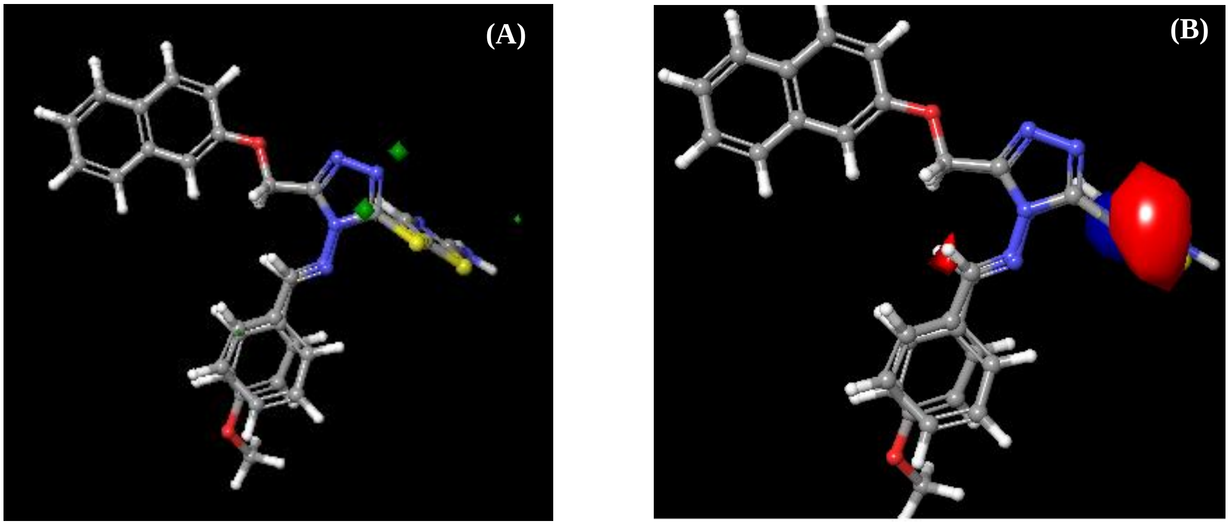

Figure S2. Field-based CoMFA model visualized in the context of favorable and unfavorable Steric (A) and Electrostatic (B) for the highest and lowest active compounds 12d and 9a, respectively.

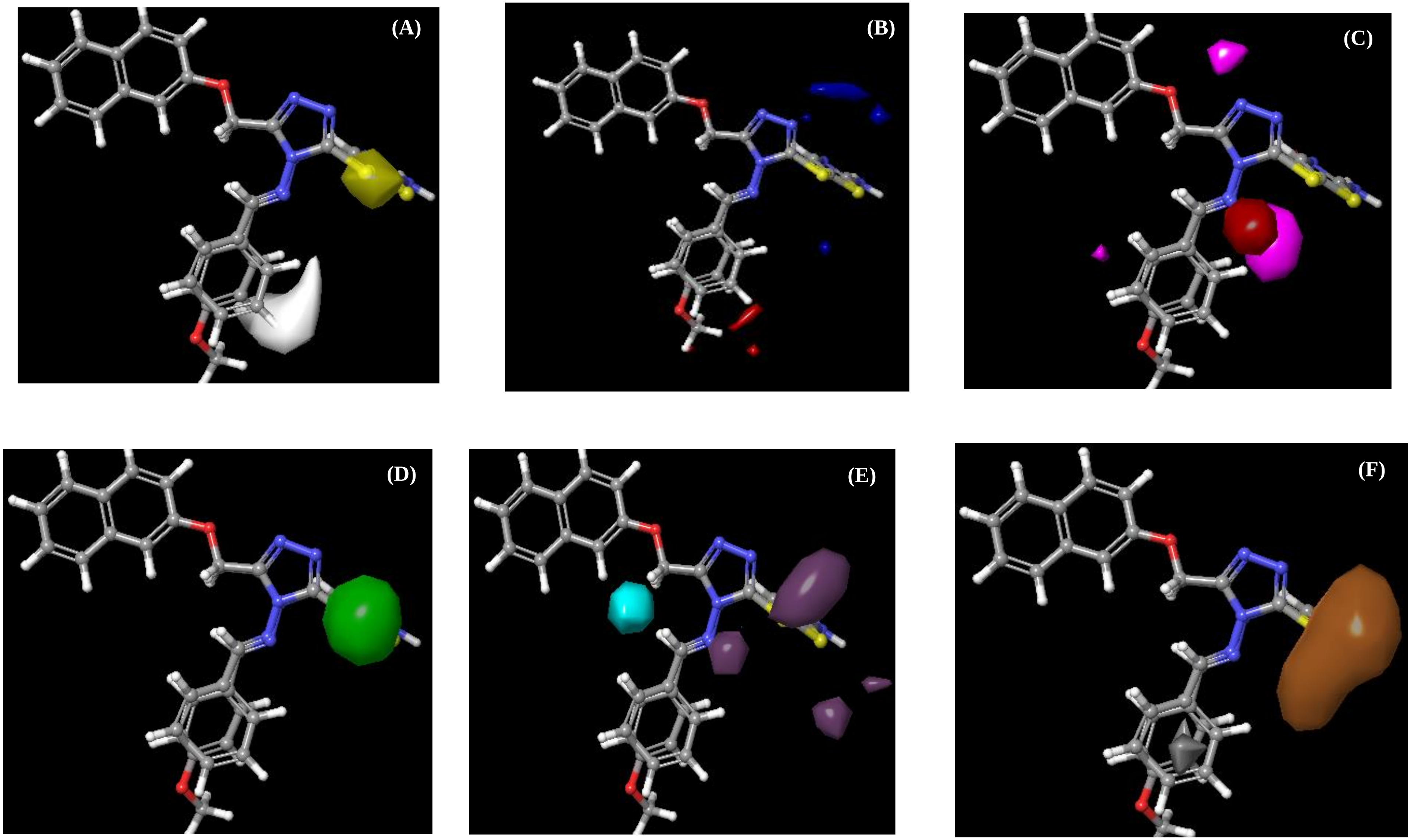

Figure S3. Field-based CoMSIA model visualized in the context of favorable and unfavorable Hydrophobic (A), Hydrogen bond donor (B), Hydrogen bond acceptor (C), Steric (D), Electrostatic (E), Aromatic (F) for the highest and lowest active compounds 12d and 9a, respectively.

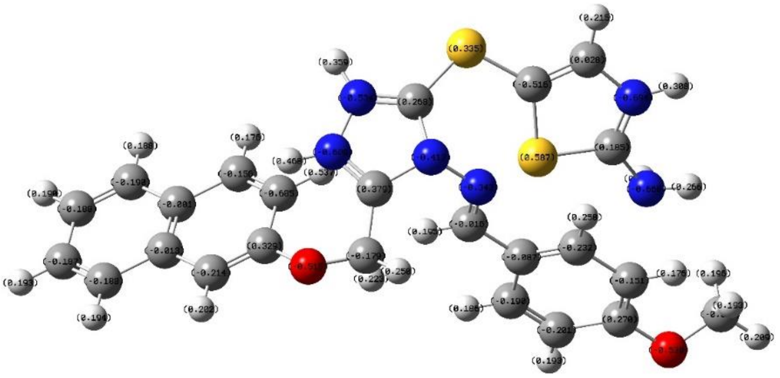

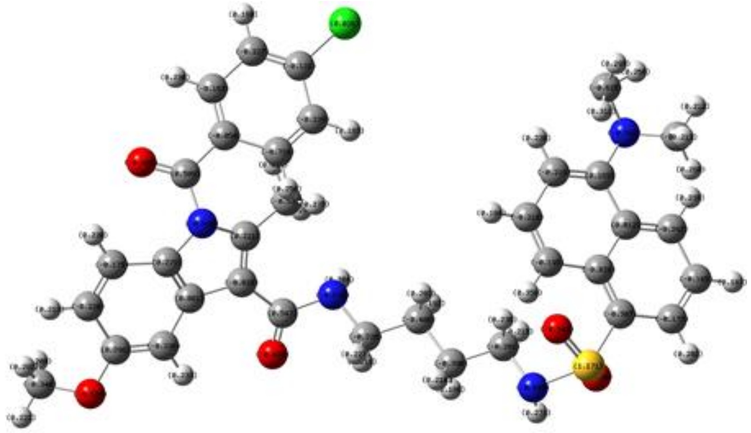

Compound 12d **Dansyl-Indomethacin**

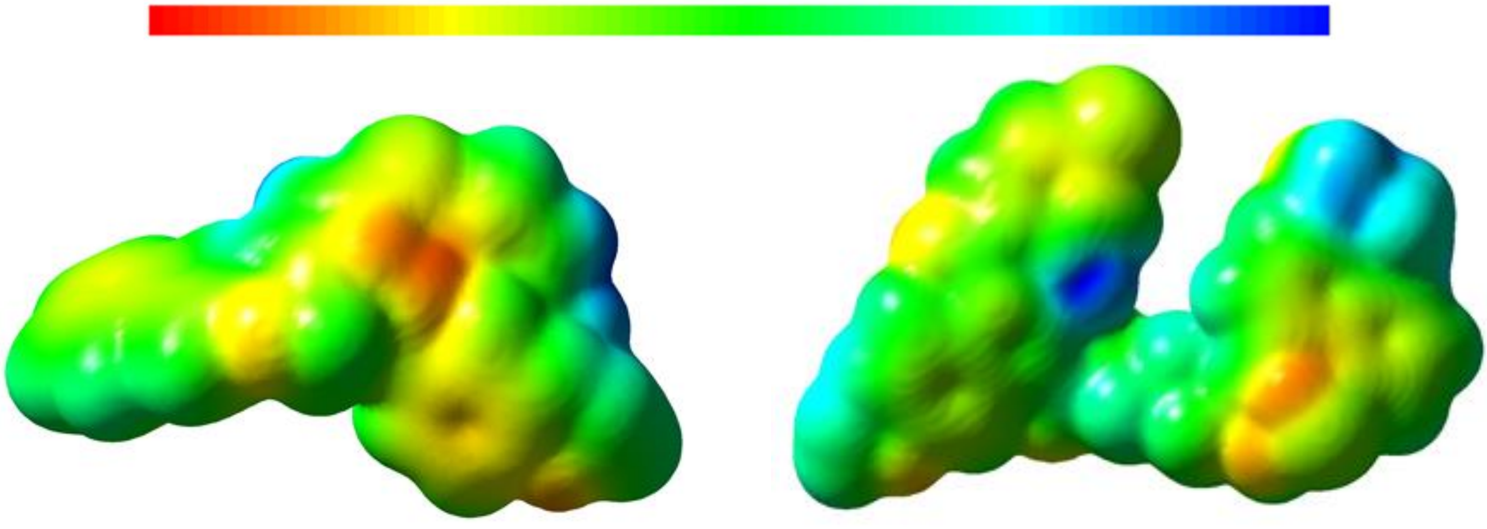

Compound 12d **Dansyl-Indomethacin**

Figure S4. (a) Mulliken charges, (b) electrostatic surface potential calculated using 3-21G* (d,p) basic set methodology (color-coded from red to blue) and density functional theory method with B3LYP functional

"These color coded isosurface values provide an indication of the overall molecular size and of the location of negative or positive electrostatic potentials. The most negative and positive electrostatic potential is colored deepest, red and blue, respectively. The intermediate shades "orange, yellow, green" indicate intermediary ranges of reactivity".

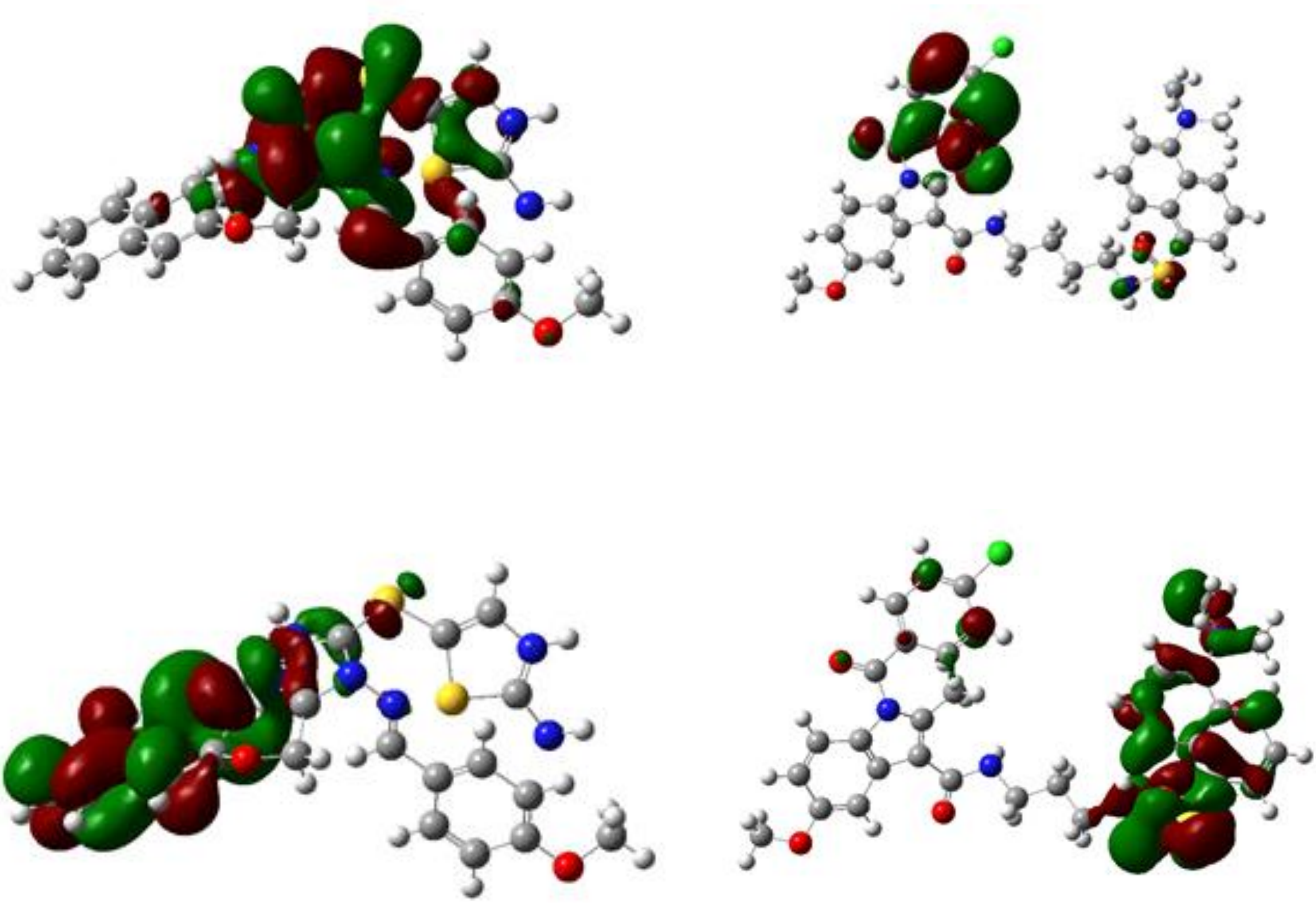

Figure S5. Plots of HOMO and LUMO of compound 12d on left side and dansyl-indomethacin on right side.

Molecular Docking

The COX-2–dansylindomethacin complex was refined for the Glide docking calculations using the protein preparation wizard applying the "OPLS-2005 force field". In the second step, water of crystallography, if present, was removed and the pH was adjusted to 7.0. In the third step, the appropriate charge and protonation state of protein were adjusted by the protein assignment script, and then the protein–inhibitor complex was subjected to energy minimization until the "RMSD" value of the nonhydrogen atoms reached "0.3 Å" to discard the steric clashes. Using ligand preparation wizard, the 3D structures of the 1,2,4-triazole derivatives were constructed and optimized with the build-panel in Maestro. Partial atomic charges were ascribed for the 1,2,4-triazole derivatives using the "OPLS-2005" force field and possible ionization states were generated at pH 7. To soften the potential for non-polar parts of the receptor, the van der Waals radii of receptor atoms were scaled by 0.8 with a partial atomic charge of 0.15. A grid box with coordinates X = 10, Y=10, and Z = 10 was generated at the centroid of the active site. Furthermore, the energy of the obtained ligand structures was minimized until it reached the RMSD cutoff of 0.01 Å

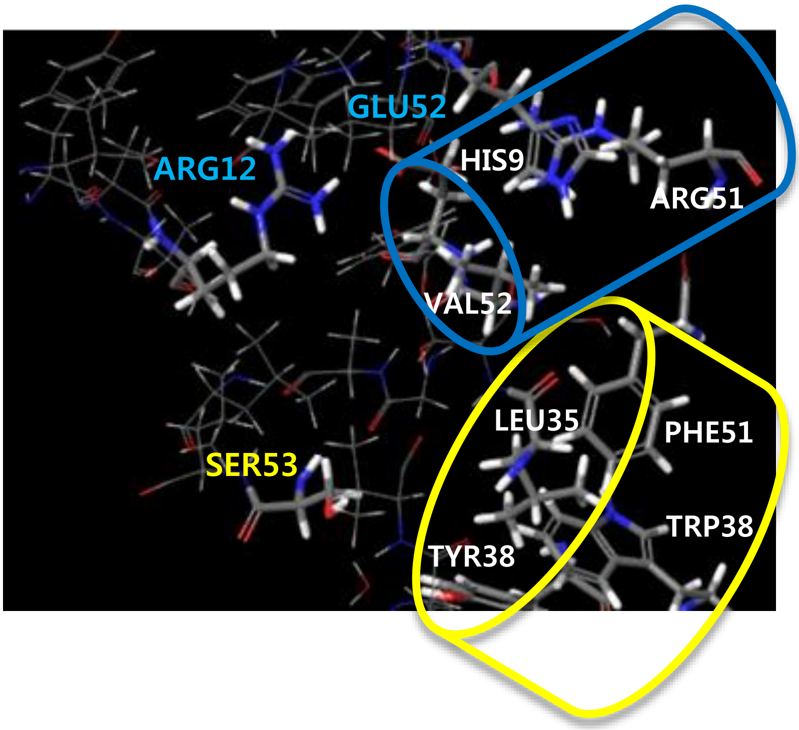

Figure S6. The binding site of COX-2 enzyme shows the secondary binding pocket in blue and the unique hydrophobic pocket in yellow.

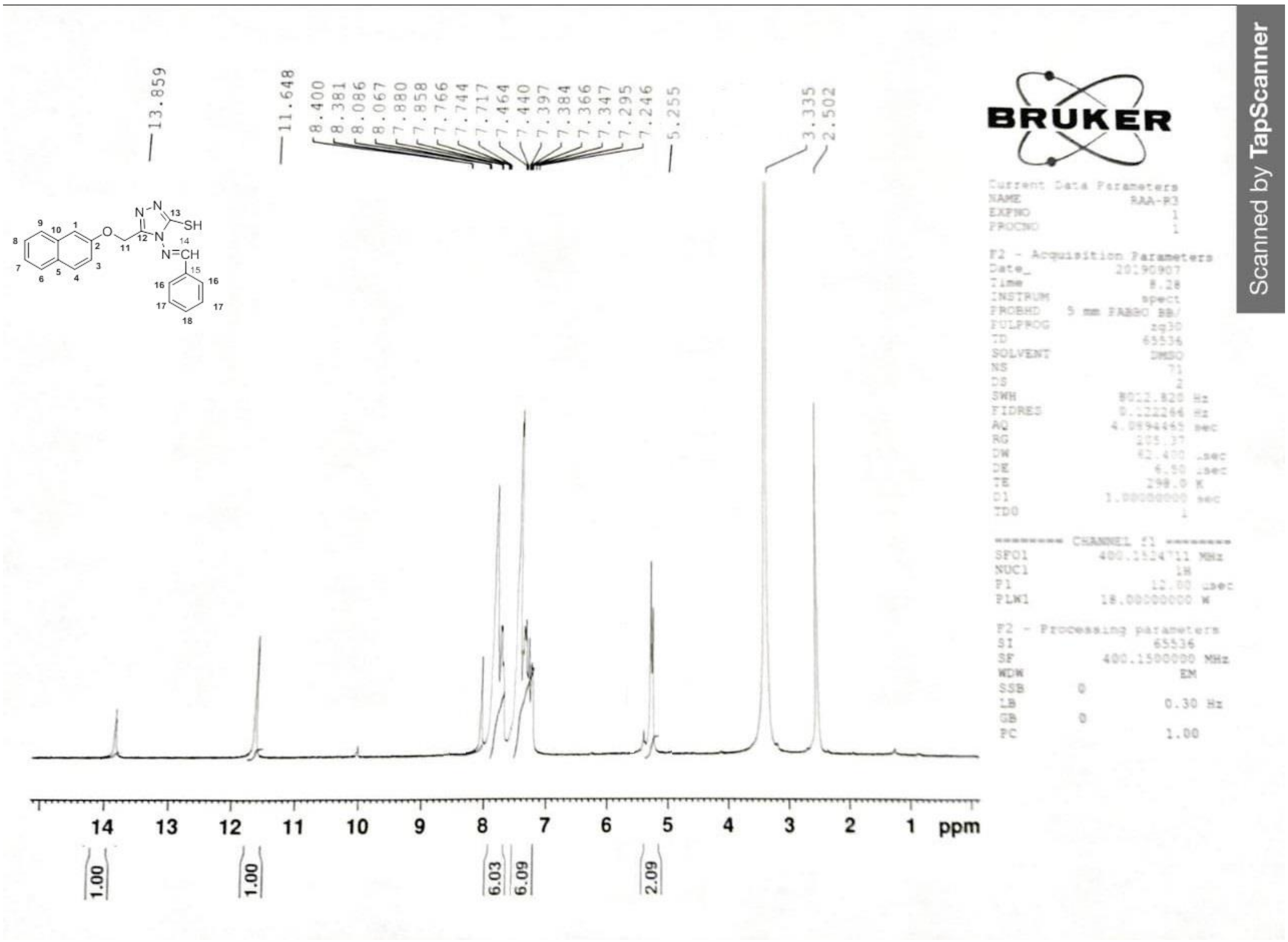

Figure S7. ^{1}H-NMR spectrum of compound 9a

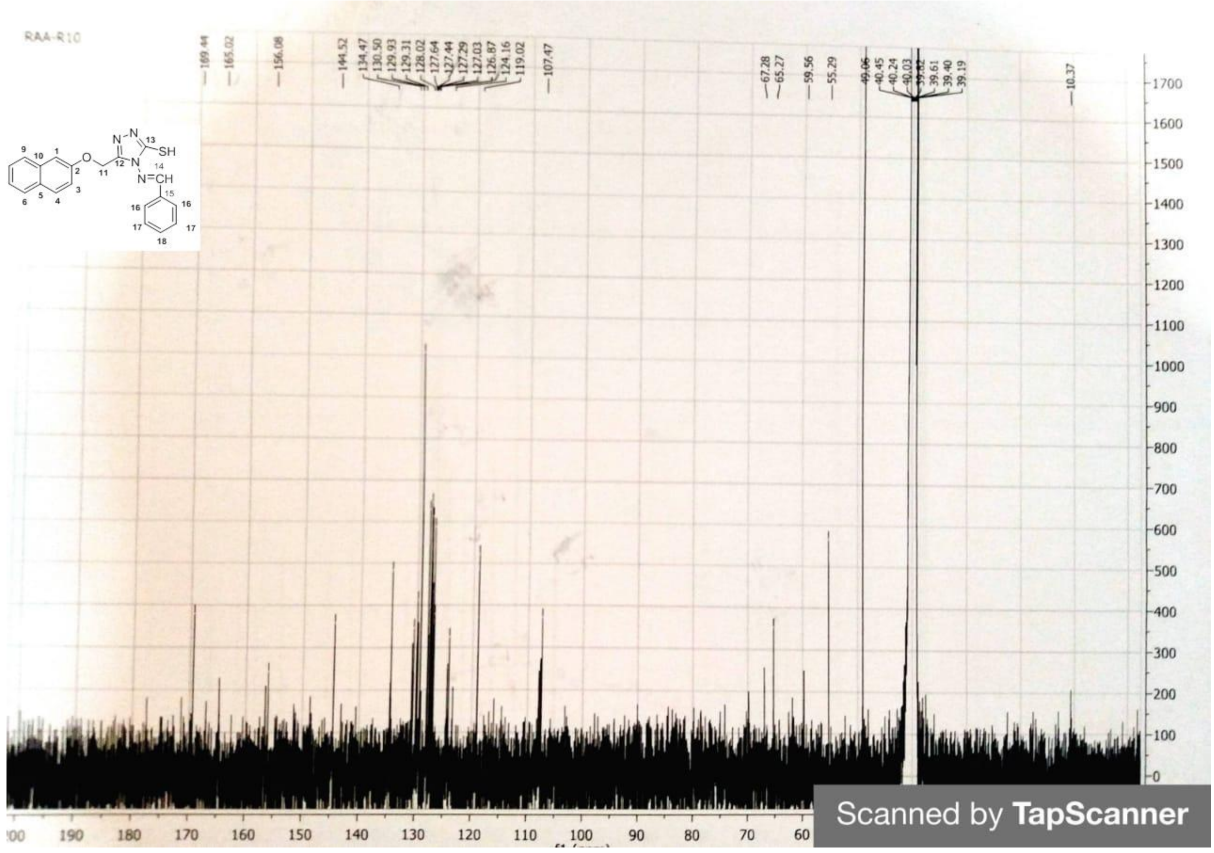

Figure S8. ^{13}C-NMR spectrum of compound 9a

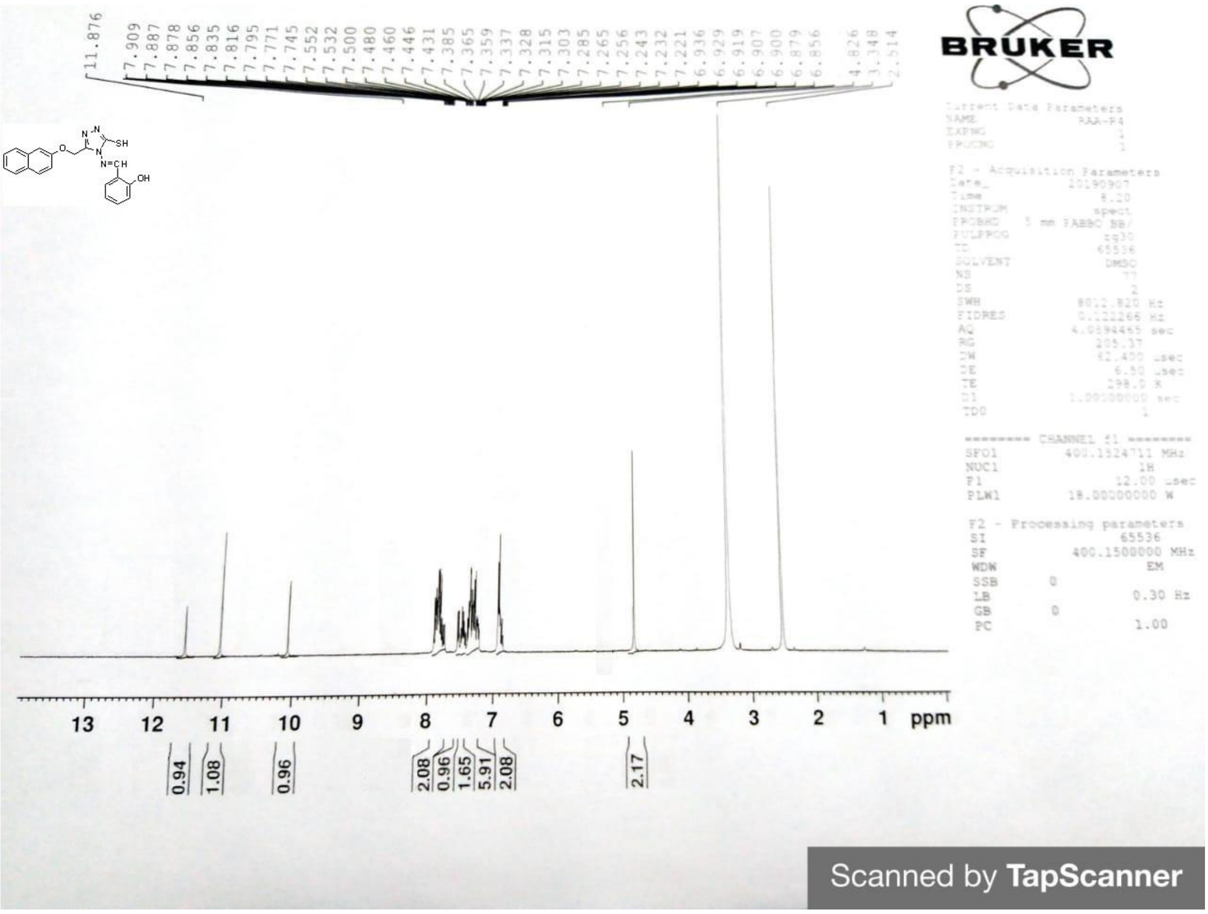

Figure S9. ^{1}H-NMR spectrum of compound 9b

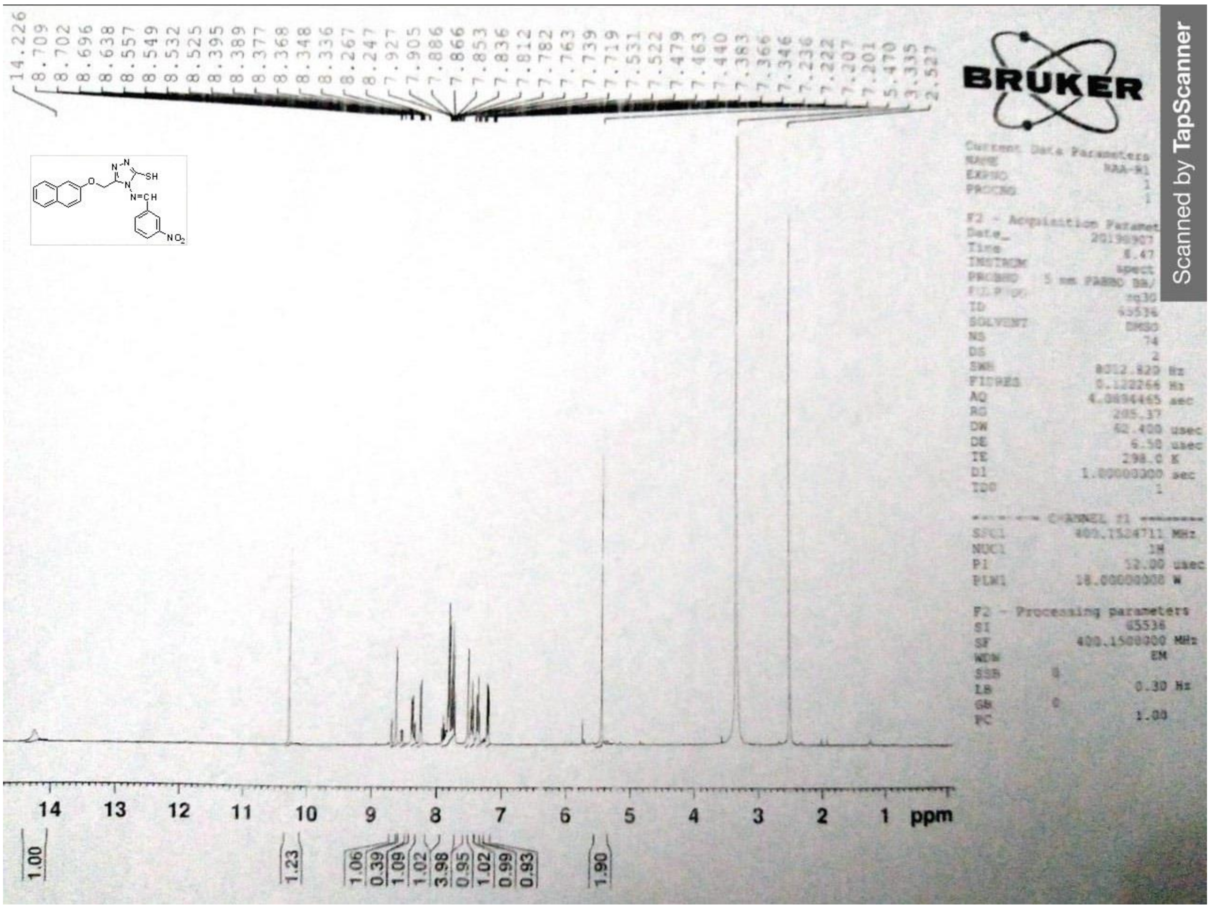

Figure S10. ^{1}H-NMR spectrum of compound 9c

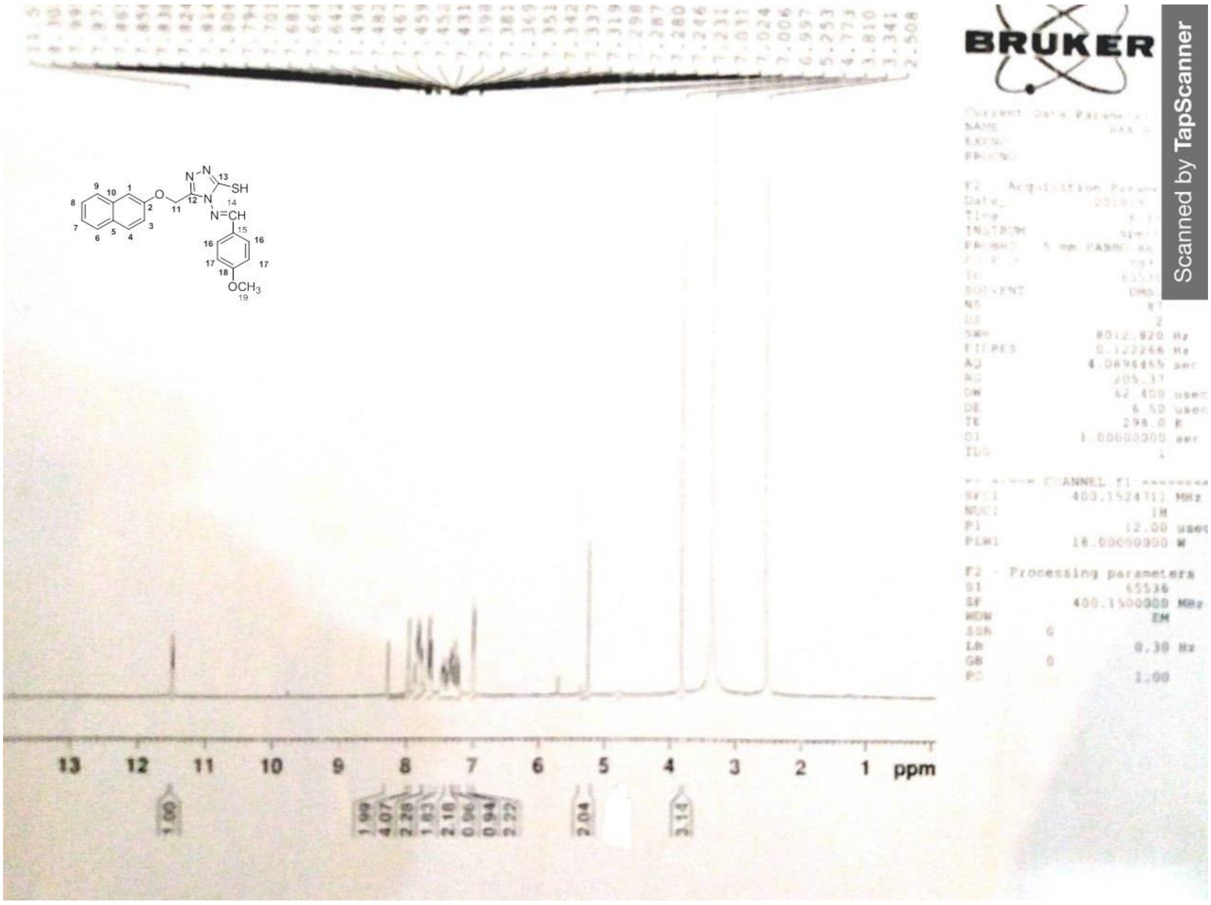

Figure S11. ^{1}H-NMR spectrum of compound 9d

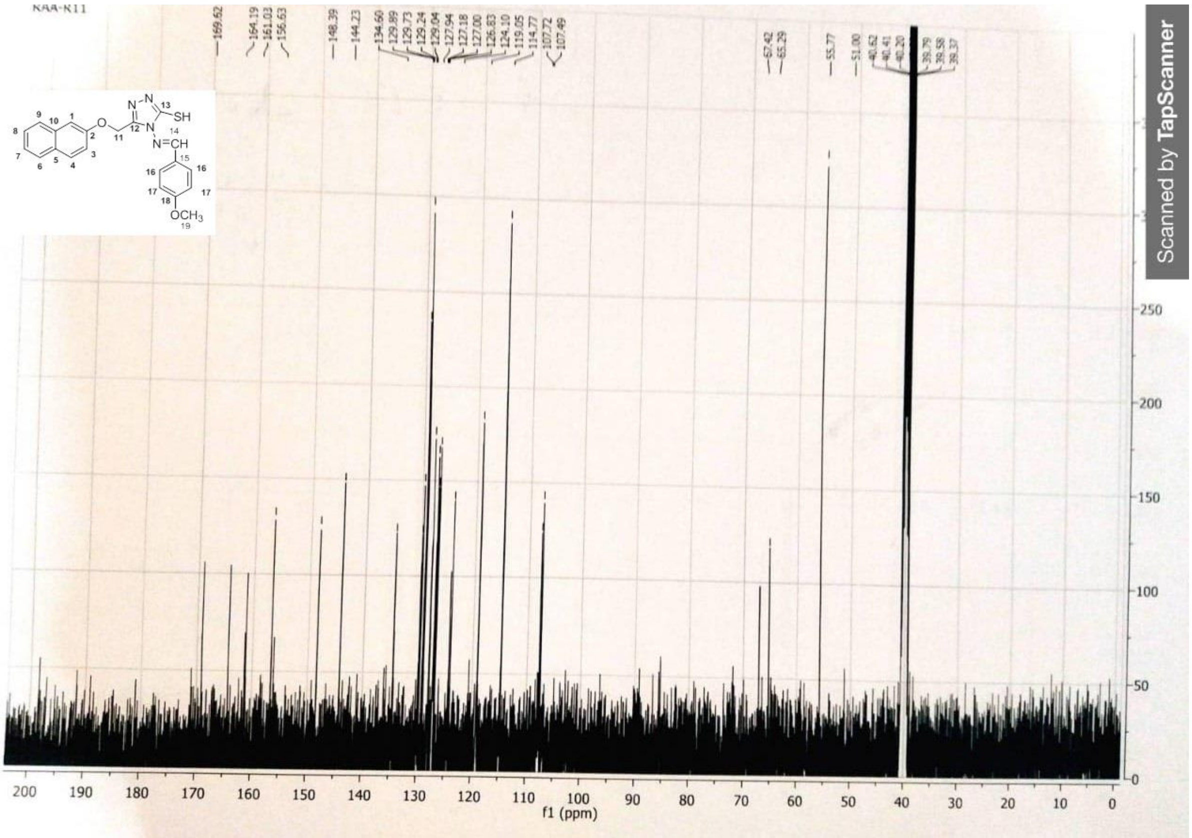

Figure S12. ^{13}C-NMR spectrum of compound 9d

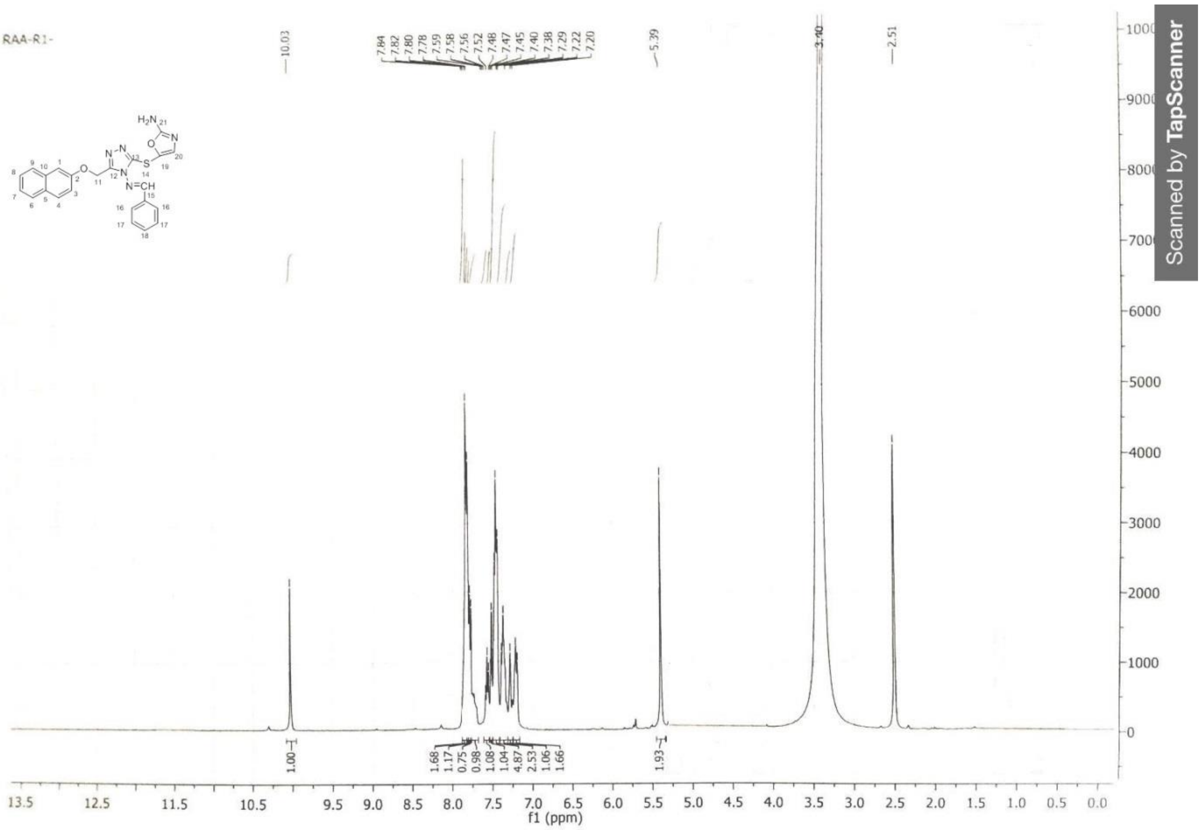

Figure S13. ^{1}H-NMR spectrum of compound 11a

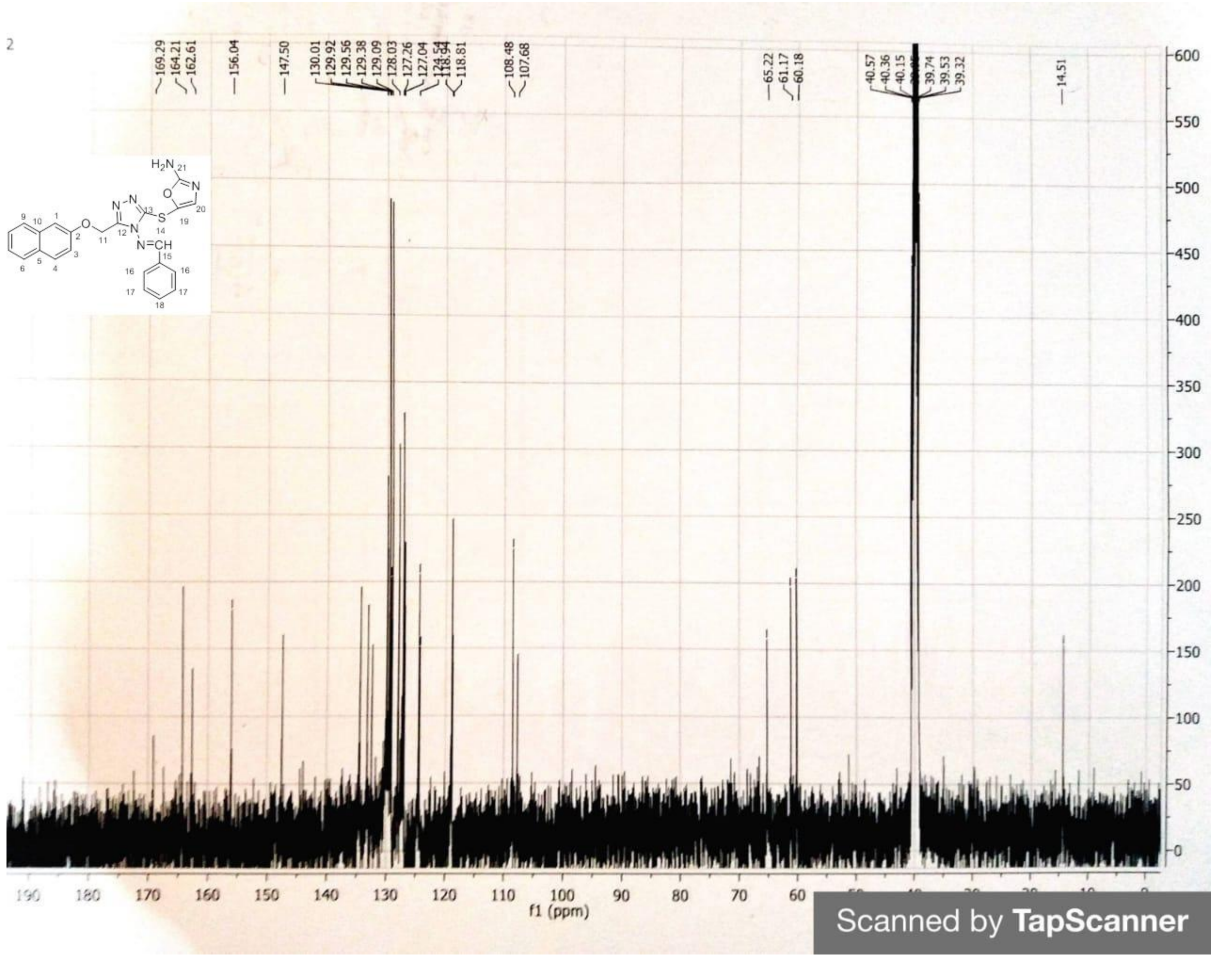

Figure S14. ^{13}C-NMR spectrum of compound 11a

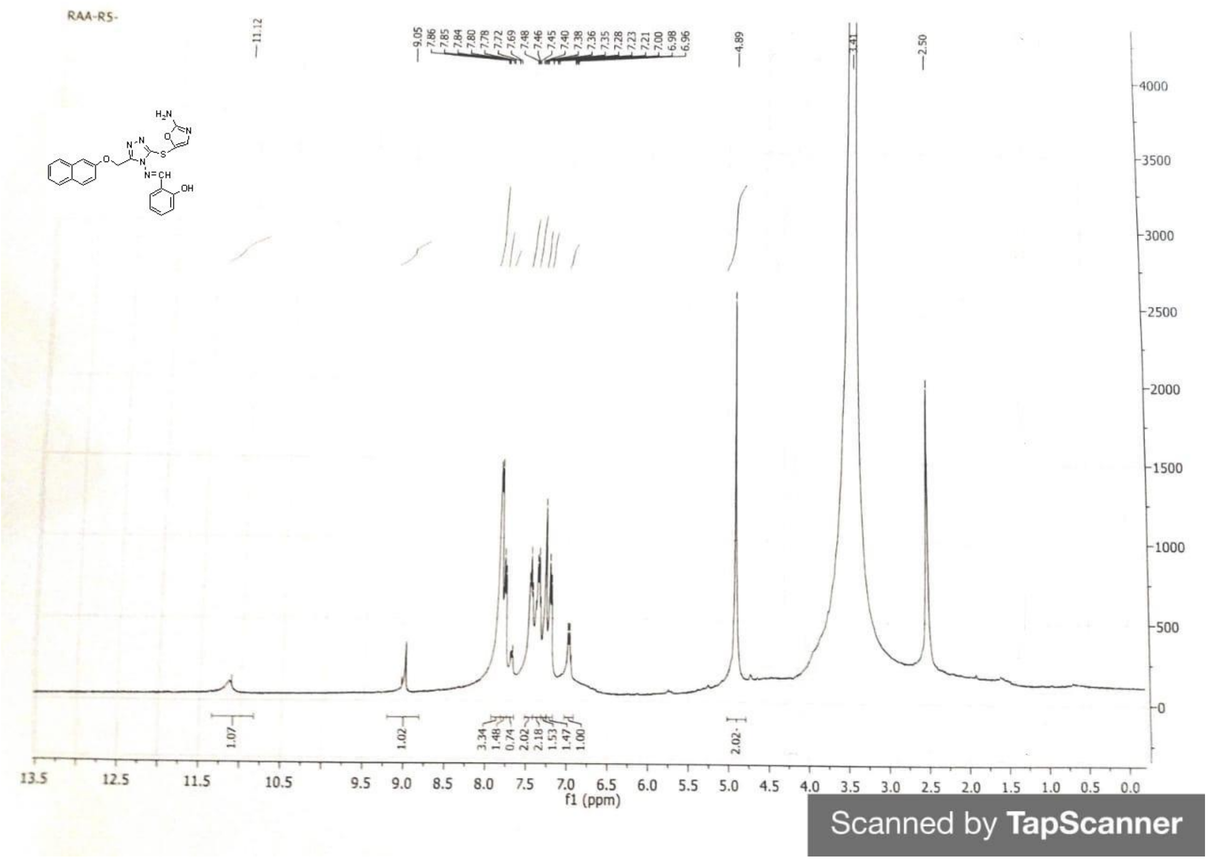

Figure S15. ^{1}H-NMR spectrum of compound 11b

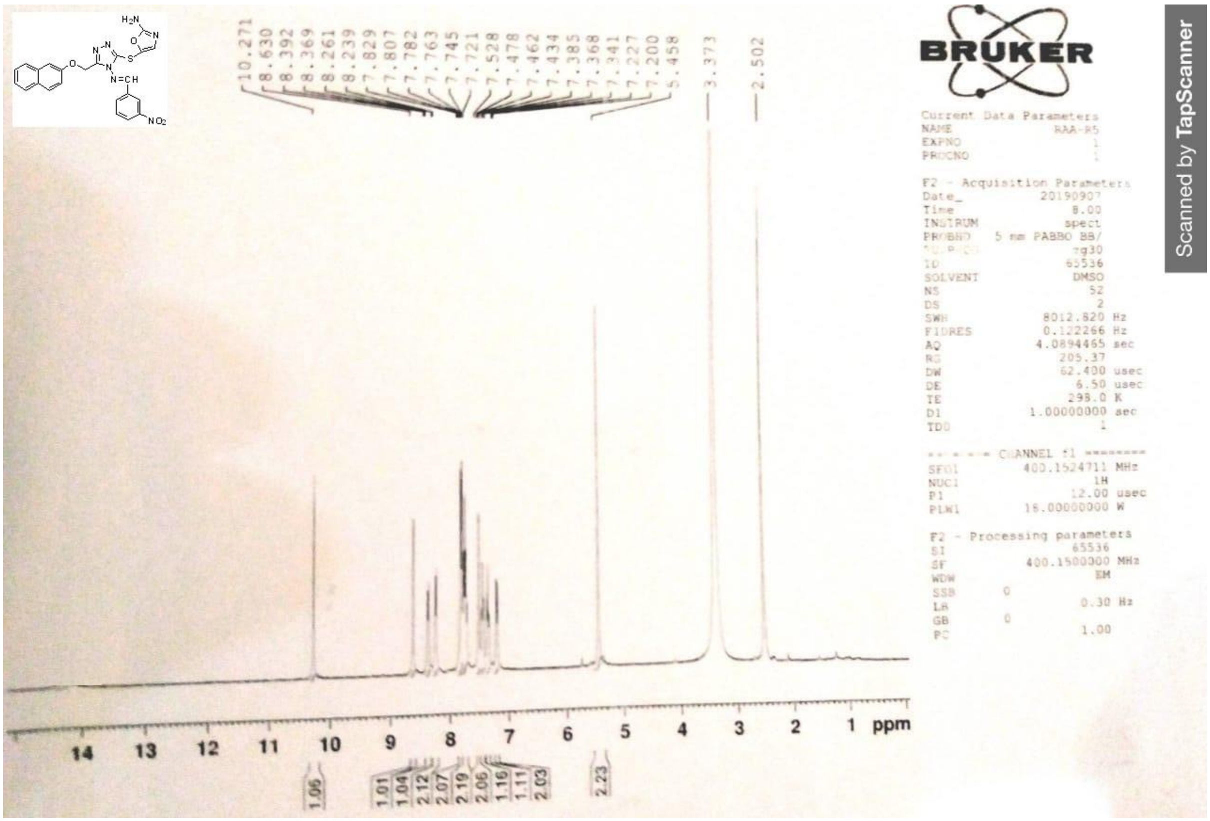

Figure S16. ^{1}H-NMR spectrum of compound 11c

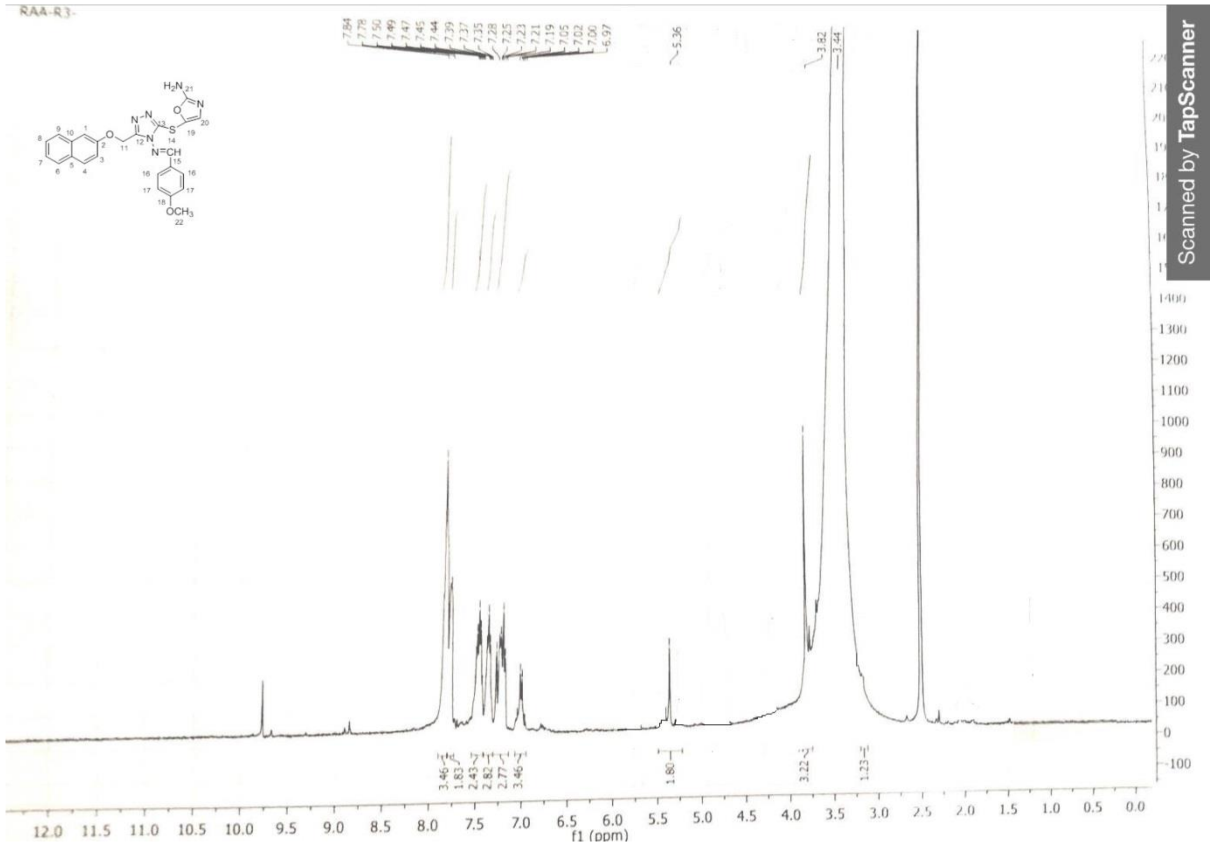

Figure S17. ¹H-NMR spectrum of compound 11d

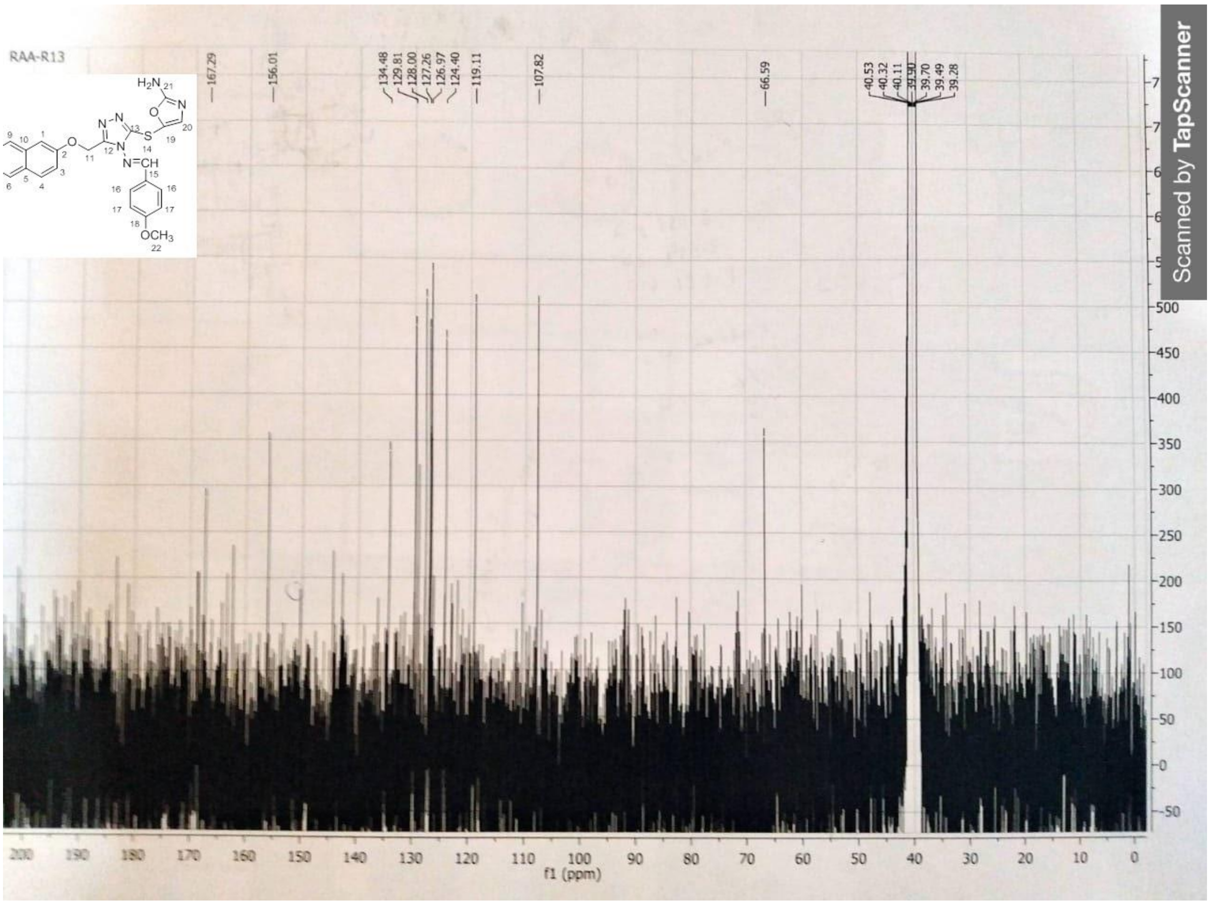

Figure S18. ^{13}C-NMR spectrum of compound 11d

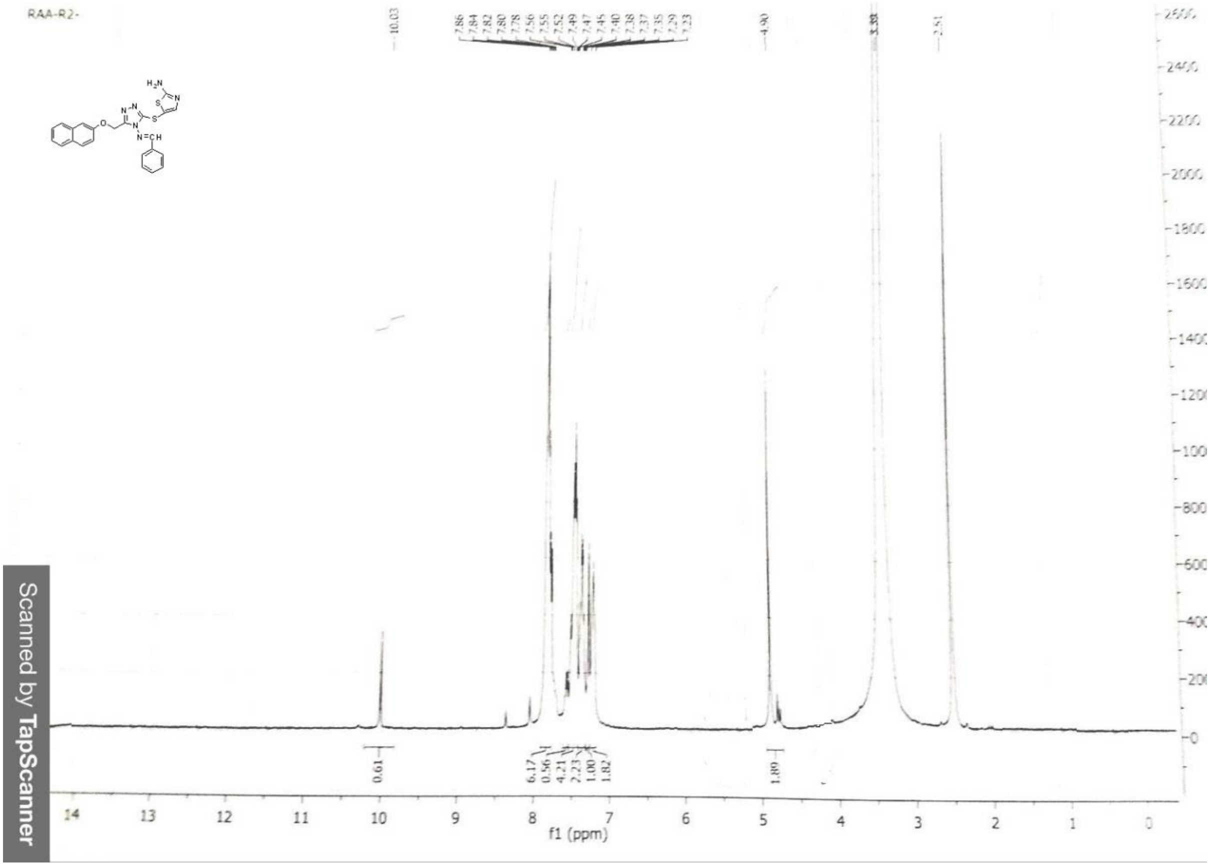

Figure S19. ^{1}H-NMR spectrum of compound 12a

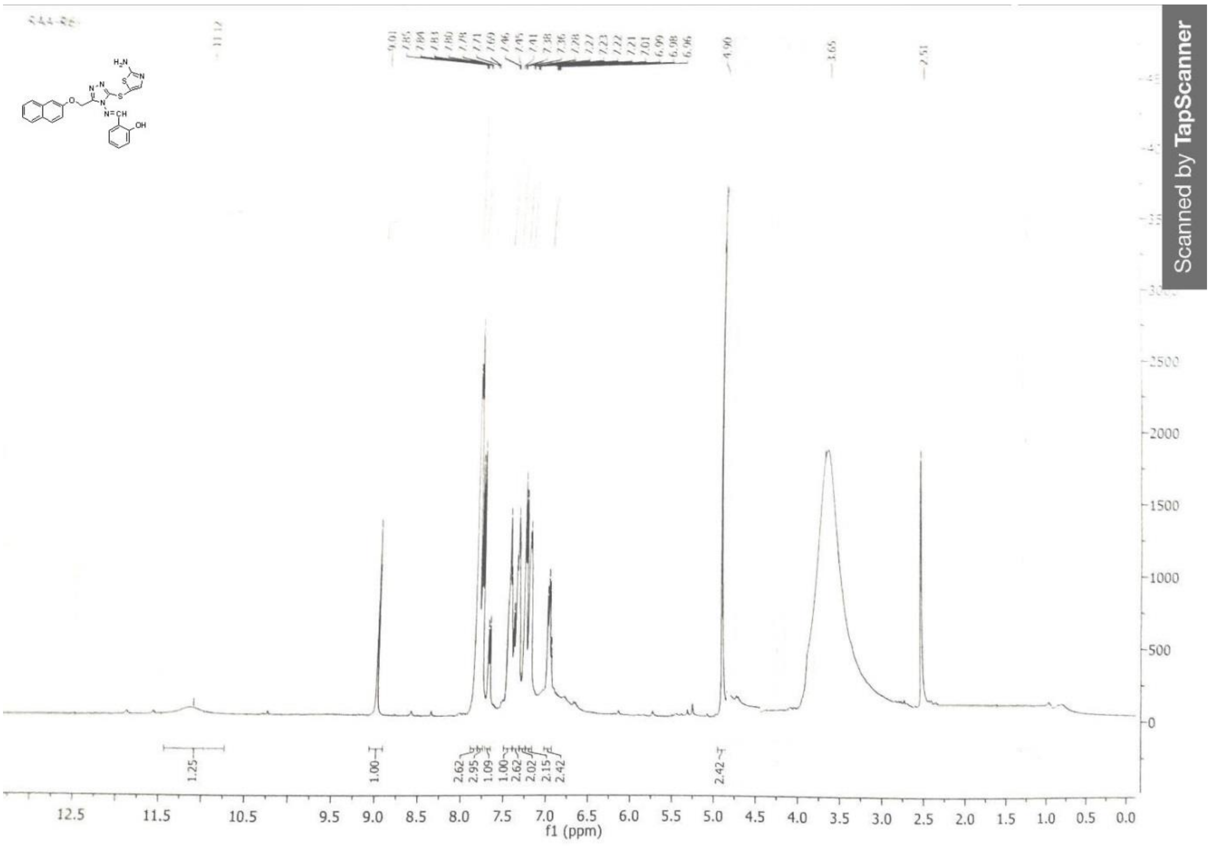

Figure S20. ^{1}H-NMR spectrum of compound 12b

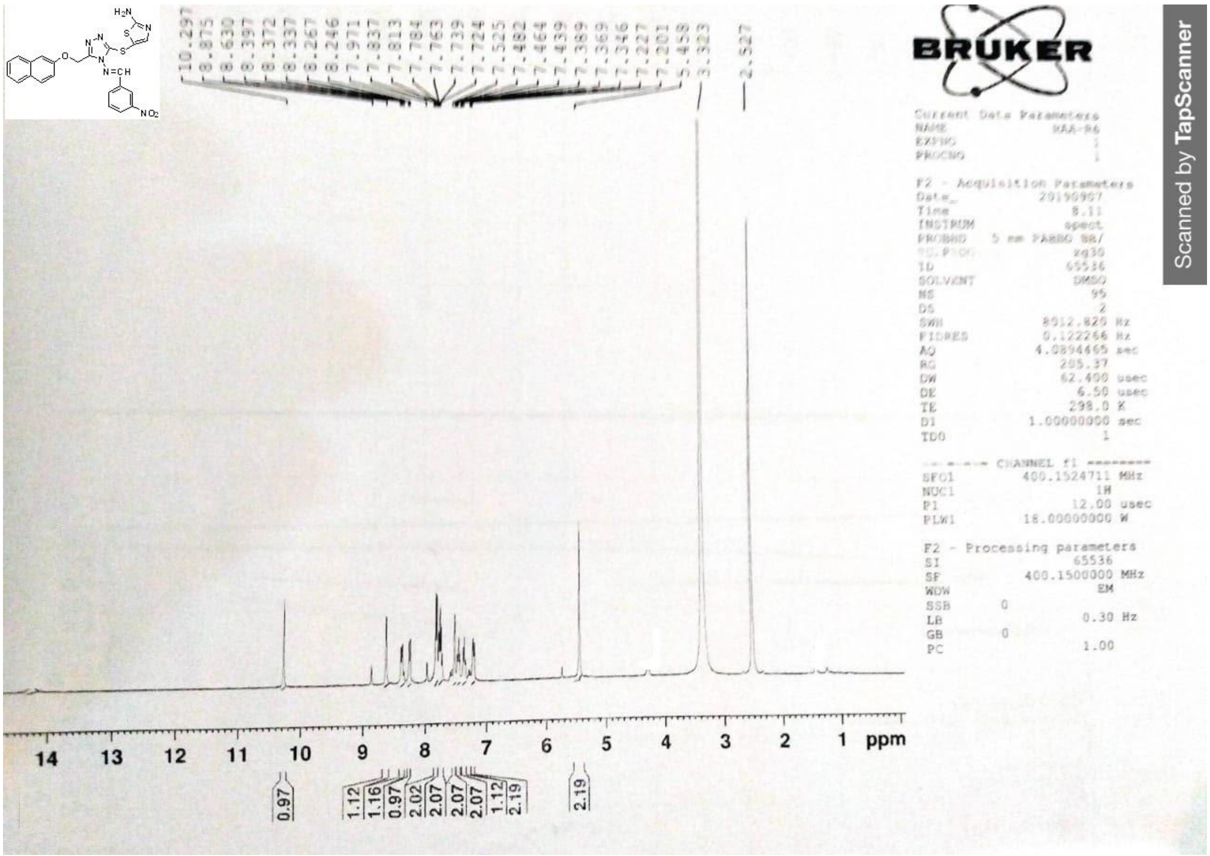

Figure S21. ¹H-NMR spectrum of compound 12c

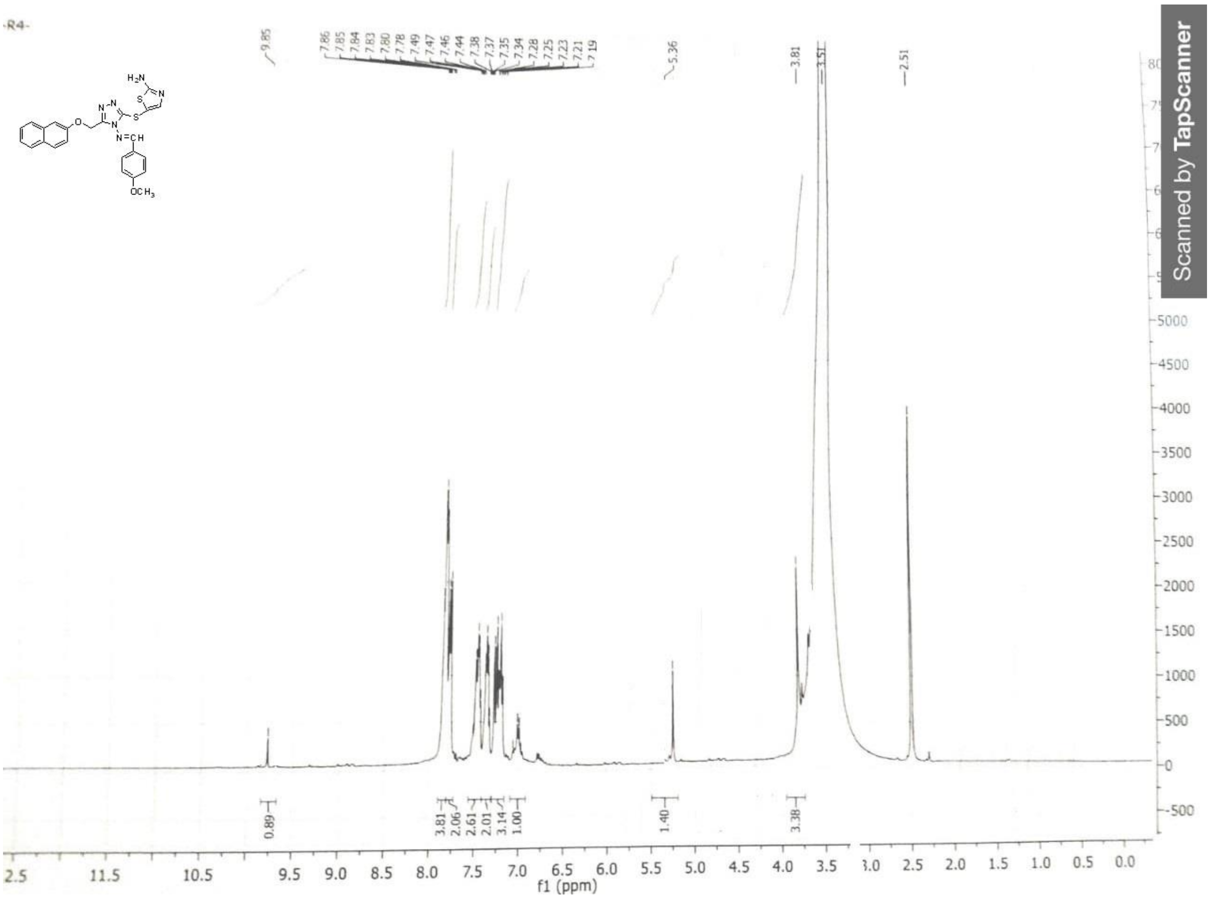

Figure S22. ^{1}H-NMR spectrum of compound 12d
